# The rice thylakoid membrane-bound ascorbate peroxidase OsAPX8 functions in tolerance to bacterial blight

**DOI:** 10.1038/srep26104

**Published:** 2016-05-17

**Authors:** Guanghuai Jiang, Dedong Yin, Jiying Zhao, Honglin Chen, Lequn Guo, Lihuang Zhu, Wenxue Zhai

**Affiliations:** 1 Institute of Genetics and Developmental Biology, Chinese Academy of Sciences, Beijing 100101, China

## Abstract

Thylakoid membrane-bound ascorbate peroxidase (tAPX) is a major H_2_O_2_-scavenging enzyme. To clarify its functions in tolerance to rice bacterial blight, we produced rice lines overexpressing and suppressing *tAPX* (*OsAPX8*). The overexpressing lines exhibited increased tolerance to bacterial pathogen. The RNA interference (RNAi) lines were considerably more sensitive than the control plant. Further analysis of the H_2_O_2_ content in these transgenic plants indicated that the H_2_O_2_ accumulation of *OsAPX8*-overexpressing plants was considerably less than that of wild-type and RNAi plants upon challenge with bacterial pathogen. Interestingly, H_2_O_2_ was the most important factor for the serious leaf dehydration and withering of rice without major resistance genes and was not the cause of hypersensitivity. It addition, wall tightening or loosening can occur according to the level of H_2_O_2_. In addition, OsAPX8 interacted with the susceptibility protein Os8N3/Xa13, and their binding repressed the reaction of OsAPX8 in tolerance to bacterial blight.

Bacterial blight, caused by *Xanthomonas oryzae* pv. *Oryzae* (*Xoo*), is one of the most destructive diseases in rice growth worldwide^1^. Rice resistance is considered the most effective and sustainable disease control method. To date, eight bacterial blight resistance genes (*Xa21*, *Xa1*, *Xa26*, *xa5*, *Xa27*, *xa13*, *Xa10* and *Xa23*) have been cloned, and their resistance molecular mechanisms are under investigation^2^,^3^,^4^,^5^,^6^,^7^,^8^,^9^,^10^. The recessive disease resistance gene *xa13* is a membrane protein of the MtN3/saliva/SWEET family and mediates a race-specific resistance to *Xoo* strain PXO99 in a manner different from that of other characterized R genes^11^. Its corresponding dominant gene, *Xa13,* is also referred to as *Os8N3/OsSWEET11* and functions as a susceptibility gene that can be transcriptionally activated by the TAL effector pthxo1 of PXO99^12^. The suppression of *OS8N3/Xa13* can confer upon rice a strong disease resistance to PXO99. Interestingly, SWEET proteins play important roles in pollen nutrition^13^ and phloem loading^14^,^15^,^16^ in addition to being involved in disease susceptibility. In addition to targeting the plasma membrane, they can localize in endogenous membranes, such as the endoplasmic reticulum (ER) and vesicular compartments^16^,^17^. These proteins generally form homo- and hetero-oligomers in these membrane compartments to create a functional pore for sugar transport. Although Xa13/Os8N3 can cooperate with COPT1 and COPT5 to promote the removal of copper from xylem vessels to provide *Xoo* with good growth conditions^18^, the rice susceptibility mechanism is highly sophisticated, and activated Os8N3 can also interact with other components to repress this resistance.

Most stress, including disease, can generate reactive oxygen species (ROS)^19^,^20^,^21^,^22^. ROS are toxic molecules that include the two free radical species superoxide anion O^−^_2_ and HO_2_, the uncharged non-radical species hydrogen peroxide (H_2_O_2_) and the highly reactive hydroxyl radical OH. The particular destructiveness of ROS depends on their reactivity. High concentrations of ROS can result in the oxidation of a variety of cellular structures, including DNA, proteins and membrane lipids; cause the disruption of metabolism; and ultimately destroy cellular structures. However, ROS are continuously generated in plant cells as the products of normal aerobic metabolism and as key signaling molecules in response to numerous developmental and environmental stimuli at low concentrations. Thus, plants have evolved a range of enzymatic and non-enzymatic antioxidant mechanisms to prevent ROS from reaching destructive levels to maintain cellular redox balance. These mechanisms involve the detoxification of ROS by carotenoids or by the action of antioxidant enzymes, such as superoxides dismutase (SOD), ascorbate peroxidase (APX), catalase, glutathione peroxidase and peroxiredoxins.

As one of the important detoxification systems, the ascorbate-glutathione cycle mediates hydrogen peroxide detoxification in chloroplasts and cytosol, of which APX is the key enzyme^23^. APXs can catalyze the conversion of H_2_O_2_ to H_2_O and O_2_ using ascorbate as the specific electron donor^24^. Tobacco plants with the suppression of cytosolic *APX1* were less tolerant of high light stress, ozone, oxidative stress and pathogen attack^25^,^26^. Interestingly, a wheat mutant with the thylakoid *APX* gene showed a 40% decrease in enzyme activity and reduced growth and photosynthetic activity when grown under controlled conditions^27^. In addition, the stripe rust resistance protein WKS1 interacts with wheat tAPX to reduce its ability to scavenge active oxygen^28^; however, attempts to suppress tobacco’s thylakoid *APX* gene have not been successful^29^. The overexpression of *APX1* or *tAPX* results in plants with enhanced tolerance to oxidative stress, suggesting that the thylakoid APX is crucial for plant survival^29^,^30^,^31^,^32^,^33^. There are eight *APX* genes in rice, which can be classified into three functional categories: cytosolic, peroxisomal and chloroplastic^34^. In addition to OsAPX3 and OsAPX6 in the peroxisomes and mitochondria, respectively, as determined by the YFP fusion protein method^35^, another APX-like protein, APX-R, targets chloroplasts and can physically interact with chloroplastic APX proteins^36^. Similarly, the rice APXs are involved in many stress reactions. For example, the overexpression of the cytosolic *APX* genes *OsAPXa*, *OsAPXb*^37^ and *OsAPX2*^38^ enhances plants’ tolerance to cold and salt stress by increasing the H_2_O_2_-scavenging capacity; deficiencies in cytosolic the APX isoforms OsAPX1/2 markedly alter the expression of genes that are associated with several key metabolic pathways, particularly the photosynthesis and antioxidant defense pathways^39^. *OsAPX8* can be up-regulated by NaCl^40^,^41^.

Although the *OsAPX8* gene has been shown to be involved in the salt stress response, few experiments have studied its molecular mechanism and signal pathway in enhancing plant resistance to bacterial pathogens. In this study, we show that the functions of *OsAPX8* in tolerance to *Xanthomonas oryzae* pv. *Oryzae* (*Xoo*) can be suppressed by Os8N3/Xa13.

## Results

### OsAPX8 is highly responsive to the induction of Xanthomonas oryzae pv. Oryzae

**Figure 1:**
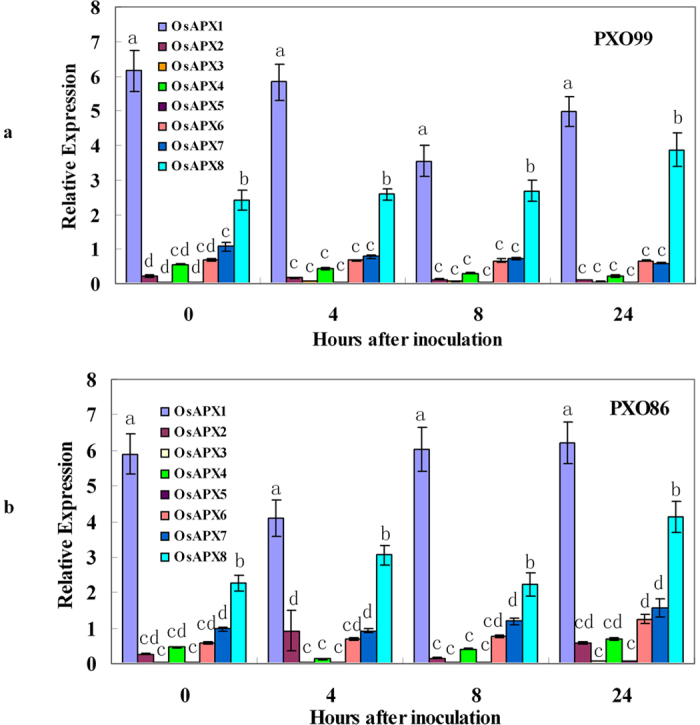
Expression of rice APX genes in leaves. (**a**) Quantitative RT-PCR analysis of the OsAPXs mRNA accumulation in TP309 plants that were challenged with *Xanthomonas oryzae* pv. *Oryzae* (*Xoo*), strain PXO99. (**b**) Quantitative RT-PCR analysis of the OsAPXs mRNA accumulation in TP309 plants that were challenged with *Xoo* strain PXO86. Leaves that were inoculated with *Xoo* were harvested at 0, 4, 8, and 24 h. The values represent the means ± SD of three replicates.

In plants, biotic and abiotic stresses can stimulate the synthesis of antioxidant molecules, such as superoxide dismutase, glutathione, ascorbate, thioredoxin, carotenoids, tocopherols, and phenolics, and enhance the activities of many antioxidant enzymes, including superoxide dismutase, catalases, and various peroxidases, such as peroxiredoxin, glutathione peroxidase, and APX^42^. There are eight superoxide dismutases, three catalases, eight peroxiredoxins, five glutathione peroxidases and eight ascorbate peroxidases (APX) in rice^34^ (http://rice.plantbiology.msu.edu/). To determine whether these antioxidant enzymes function in resistance to *Xanthomonas oryzae* pv. *Oryzae* (*Xoo*), we examined their expression in the *japonica* rice cultivar TP309 challenged with two *Xoo* races: PXO99 and PXO86. Previous studies have shown that PXO99 and PXO86 are the main *Xoo* races for the identification of major disease resistance genes, such as *Xa21*, *xa13*, Xa27, *xa5*, *Xa26*, *Xa10* and *Xa23*. The transcript accumulation patterns of the 32 gene*s* were analyzed 4, 8 and 24 h after inoculation at the tillering stage. RT-qPCR analysis revealed that the expression of one superoxide dismutase gene, LOC_Os05g25850; two catalase genes, LOC_Os02g02400 and LOC-Os03g03910; and one peroxiredoxin gene, LOC_04g33970, was induced only by PXO99 (Supplementary Fig. S1), whereas the expression of *OsAPX2*, *OsAPX4*, *OsAPX6* and *OsAPX7* was slightly induced only by PXO86 (Fig. 1a). Two glutathione peroxidase genes, LOC_Os06g08670 and LOC_Os04g46960, were initially suppressed and then induced by PXO99 after 8 h (Supplementary Fig. S1). Interestingly, *OsAPX8* expression was specifically induced by either PXO99 or PXO86, and its transcript accumulation was increased by approximately 2-fold at 24 h compared to the control. Other genes, such as *OsAPX1*, *OsAPX3*, *OsAPX5* and LOC_Os06g51150, LOC_Os01g16152, LOC_Os01g48420, LOC_Os04g33970, LOC_Os06g09610, LOC_Os06g42000, and LOC_Os07g15670, and the majority of the superoxide dismutase genes did not respond to *Xoo* (Fig. 1 and Supplementary Fig. S1). These results indicate that *OsAPX2*, *OsAPX4*, *OsAPX6*, and *OsAPX7* and LOC_Os05g25850, LOC_Os02g02400, LOC-Os03g03910, and LOC_04g33970 might be involved in the induced defense responses that are trigged by PXO86 and PXO99, respectively; only *OsAPX8* is highly responsive to both PXO86 and PXO99 and is likely involved in basal defense. We chose the *OsAPX8* gene for further analysis.

### OsAPX8 is expressed throughout the plant and targets chloroplasts

**Figure 2:**
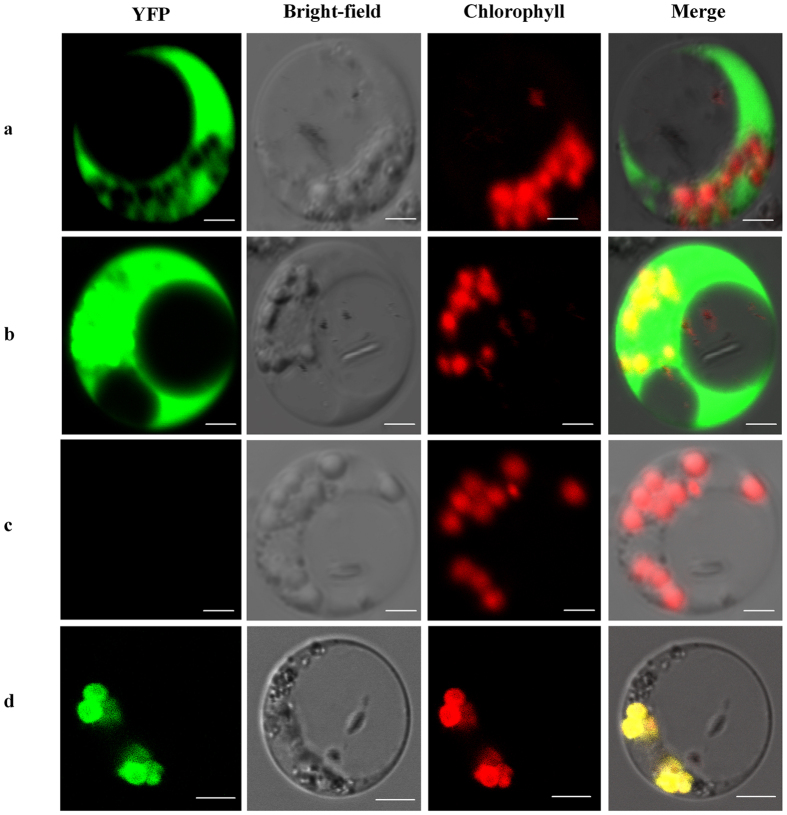
Subcellular localization of OsAPX8. Transient expression in TP309 protoplasts demonstrated that OsAPX8 targets the chloroplast. Chloroplasts were visualized by red chlorophyll autofluorescence and yellow YFP-OsAPX8. (**a**) Images of 35S::CPK17G2A-NES-YFP-transformed protoplasts. The cytosolic localized YFP fusion protein CPK17G2A-NES-YEF was used as a cytosolic localization marker. (**b**) Images of 35S::YFP-transformed protoplasts. (**c**) Images of non-transformed protoplasts. (**d**) Images of 35S::YFP-OsAPX8-transformed protoplasts. The scale bar is 2 um.

OsAPX8 possessing a C-terminal hydrophobic region that is characteristic of transmembrane domains is presumed to be a putative thylakoid-membrane-bound isoform (Supplementary Fig. S2)^34^; however, the subcellular localization of OsAPX8 has not been directly verified. This gene is expressed highly in the shoot, leaf and young panicle but at low levels in the root (Supplementary Fig. S3). Although the expression of *OsAPX8* in the root was up-regulated by NaCl^40^, this level is considerably lower than that in other tissues under salt treatment. We measured the transient expression of the OsAPX8-YFP fusion protein under the control of the CaMV 35S promoter in TP309 protoplasts. The protoplasts non-transformed or transformed with empty vector (35S::YFP) and 35S::CPK17G2A-NES-YFP, the cytosolic localized YFP fusion protein construct^43^ were used as controls. The yellow fluorescence of OsAPX8-GFP was observed in chloroplasts and overlapped well with the red autofluorescence of chloroplast (Fig. 2), indicating that OsAPX8 is a chloroplast-localized protein.

### Generation of transgenic OsAPX8 overexpression and RNAi lines

**Figure 3:**
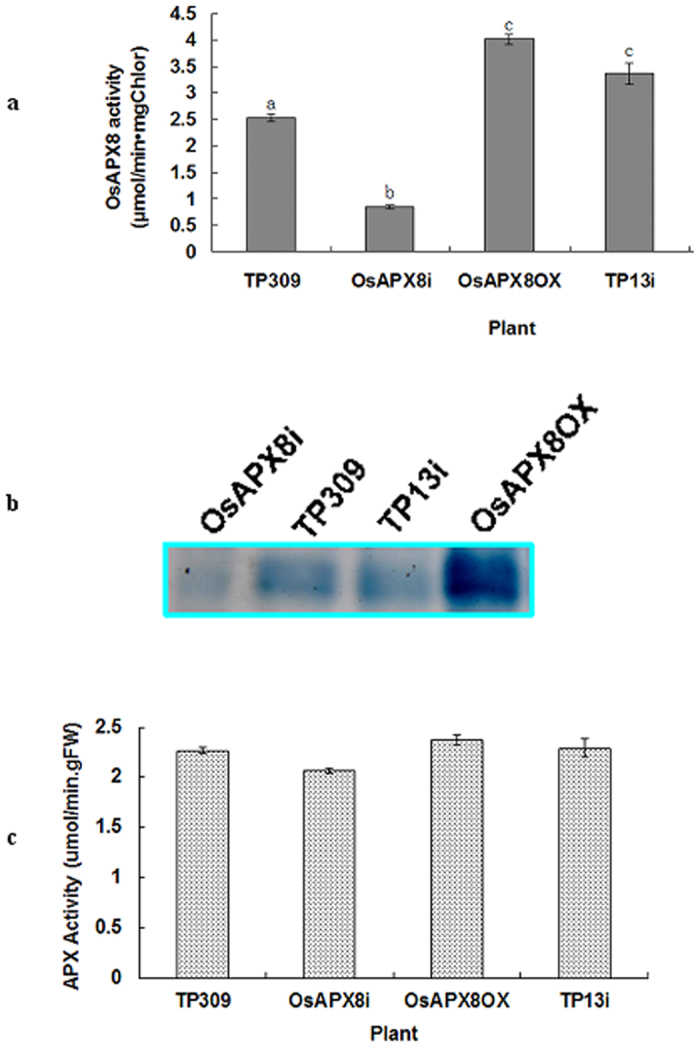
Molecular and biochemical characterization of transgenic plants with OsAPX8 overexpression or RNAi. (**a**) The OsAPX8 enzymatic activity was measured spectrophotometrically in transgenic TP309 plants with OsAPX8-OX (J25-3), OsAPX8i (J36-1) or OS8N3/Xa13 RNAi (TP13i). (**b**) Staining of tAPX activity on native PAGE gels. The solubilized fractions containing 500 μg of protein from the thylakoidal extracts of TP309, OsAPX8i, OsAPX8OX and TP13i transgenic lines were loaded in each lane. The staining of APX activity was performed as indicated in the experimental procedures. (**c**) The total APX enzymatic activity was measured spectrophotometrically in these transgenic plants. Each value is ± SD of three replicates. Different letters **(a–c**) indicate significant differences (P < 0.05) between lines.

Previous studies have shown that the *OsAPX8* was initially repressed and subsequently regulated by salt stress^35^,^40^. To determine whether there is a relationship between stress tolerance and the expression level of *OsAPX8*, we constructed the *OsAPX8* overexpression (OsAPX8-OX) and RNAi silence (OsAPX8i) binary plasmids and transferred them to the rice variety TP309 to yield 20 and 15 hygromycin-positive independent T_0_ lines, respectively. No difference was observed in the growth or morphology among the control and transgenic plants under normal growth conditions. To determine the expression levels of *OsAPX8* in these transgenic plants, these T_0_ plants were analyzed through quantitative RT-PCR using a pair of *OsAPX8*-specific primers. At least 5 independent lines with the OsAPX8-OX plasmid showed that the expression of *OsAPX8* mRNA was increased by three-fold more than that in the transformed TP309 containing the p1300 plasmid, and 11 suppression lines showed an expression reduction of approximately 70–100% (data not shown). The APX activities of these transgenic plants were further analyzed. The tAPX activity was approximately 70% lower in OsAPX8i plants and 60% higher in OsAPX8-OX plants compared to control plants (Fig. 3a). This result was further confirmed by analyzing the tAPX activity in native PAGE (Fig. 3b). However, no significant differences in the total APX activity were detected among these plants when grown under common culture conditions (Fig. 3c). In addition, the tolerance of T1 plants from these transgenic lines was tested. Four OsAPX8-OX lines (J25-2, J25-3, J25-4 and J26-4) and three OsAPX8i lines (J38-1, J38-3 and J38-4) were planted on 1/2 MS-agar plates that were supplemented with different concentrations of NaCl, and the germination was measured and statistically treated. No discernible change in germination force was observed in the presence of up to 200 mM NaCl. However, germination was significantly reduced in the presence of 250 mM or higher concentrations of NaCl; in particular, the OsAPX8i seeds were hypersensitive (Supplementary Table S1). For the physiological characterization of the seedlings, the seedlings were transferred to culture glasses containing ½ MS agar with different concentrations of NaCl after germination. After 20 days, the seedlings on medium containing 200 mM and 250 mM NaCl grew normally except that some leaves turned yellow, whereas those that grew on medium containing high NaCl (300 mM NaCl) showed a different phenotype. Although the roots of these seedlings were suppressed, the OsAPX8-OX plants largely survived, whereas the control and OsAPX8i plants could not survive at 300 mM NaCl (Supplementary Fig. S4). These results indicate that we obtained the OsAPX8i and OsAPX8-OX transgenic plants in the TP309 background. Similarly, we constructed transgenic lines with the two plasmids in the Nipponbare background (data not shown).

### OsAPX8 improves tolerance to bacterial blight in rice plants

**Figure 4:**
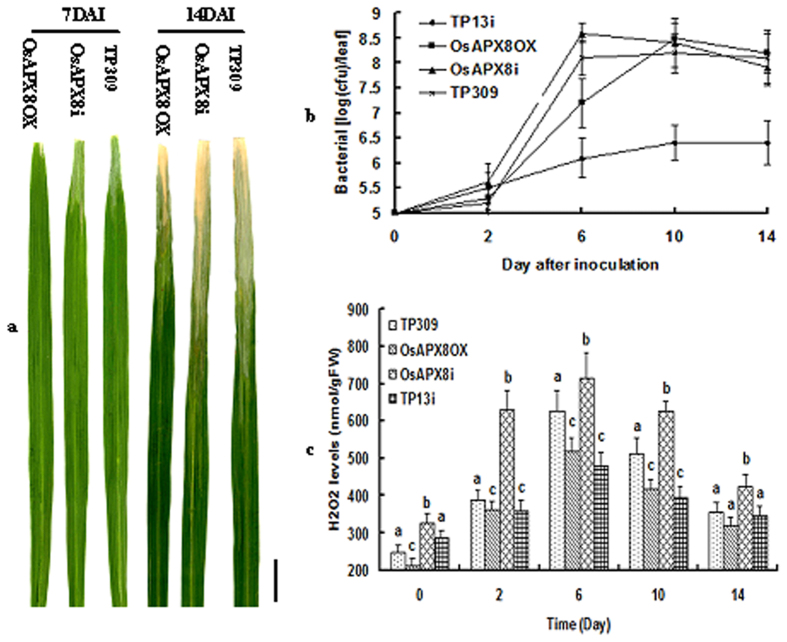
Bacterial blight tolerance analysis in OsAPX8 transgenic plants. (**a**) OsAPX8OX plants showed a stronger tolerance to the pathogen in the early stage (<7 days) and were as susceptible as TP309 and OsAPX8i plants later (7–14 days). Photograph of rice leaves showing lesion development taken one or two weeks after inoculation with PXO99. (**b**) Growth of PXO99 in the leaves of OsAPX8 transgenic plants and control lines. (**c**) The H_2_O_2_ contents in the OsAPX8 transgenic plants and control lines (TP309 and TP13i) were analyzed after inoculating with PXO99. The values are the means ± S.D. for at least three independent experiments. Different letters (**a**–**c**) indicate significant differences (P < 0.05) between lines. The scale bar is 1.2 cm.

**Figure 5:**
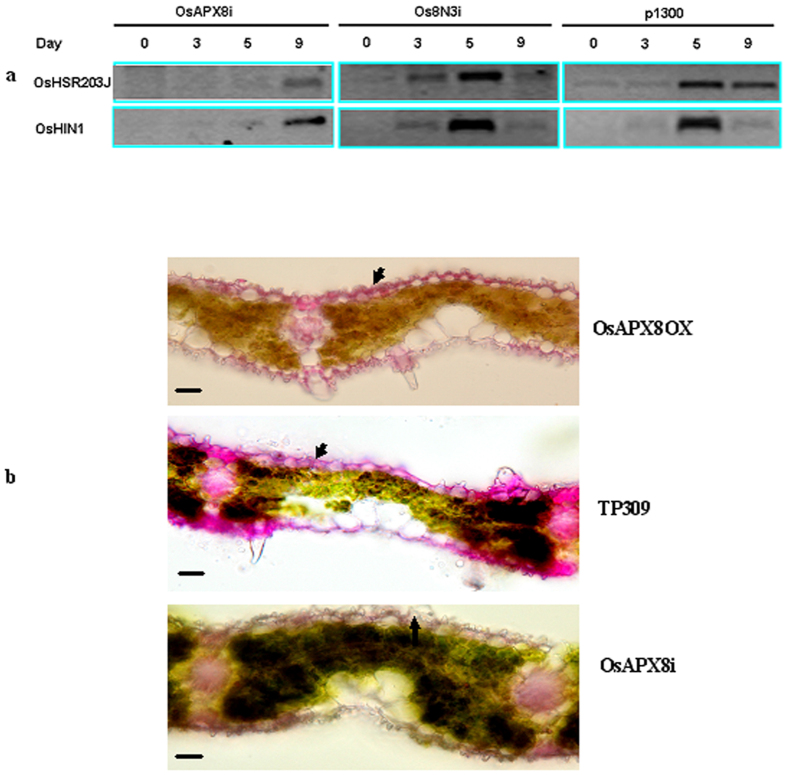
Effect of overproduced H_2_O_2_ on leaf cells. (**a**) The expression of the hypersensitive response (HR) marker genes OsHSR203J and OsHIN1 in transgenic plants. (**b**) The transverse sections of leaves of transgenic plants. Leaves were taken one week after inoculation with PXO99. The scale bar is 50 μm.

We examined the resistance phenotype of OsAPX8 T_2_ transgenic plants from four OsAPX8-OX lines and three OsAPX8i lines. These plants were planted in a greenhouse and inoculated with the PXO99 of *Xoo* at the tillering stage, after which the disease symptoms were characterized. The lesions of OsAPX8-OX transgenic plants were initially suppressed and developed more slowly than did those of wild-type plants after inoculation until day 7. Then, the lesions developed rapidly and were not different from those of the control plants. The OsAPX8Ri plants showed a different phenotype. The inoculation site of the OsAPX8Ri leaf began to dehydrate and wither on day 2 and turned to grayish-green rapidly, whereas that of the control plant withered on day 4, and the lesions were shorter. A bacterial growth curve analysis confirmed that the *Xoo* levels were higher in the OsAPX8i lines than in the OsAPX8-OX line and control line TP309 (Fig. 4a,b). Interestingly, the lesions of the transgenic plants in the Nipponbare background developed later than did those in TP309 for approximately 2–3 days (data not shown). These results indicate that the knockdown of OsAPX8 may affect the basal levels of resistance to *Xoo* in rice. In addition, PXO99 caused the inoculation site of rice leaf to experience serious dehydration and withering and to turn grayish-green instead of causing the necrotic hypersensitivity effect in both transgenic plants and TP309. In addition, these leaves inoculated with *Xoo* folded over lengthwise just below the cut surface. This result is distinct from the hypersensitivity of the Os8N3Ri/TP13i plant, a RNAi transgenic line of Os8N3/Xa13^8^,^12^. The H_2_O_2_ content analysis showed that this value increased more rapidly in OsAPX8Ri plants than in OsAPX8-OX and TP309 plants with empty vector and led to the serious dehydration of leaf cells (Fig. 4c). These results indicate that plants that were challenged with *Xoo* mainly produced H_2_O_2_ and that the ascorbate-glutathione pathway, especially tAPX, is a key H_2_O_2_-removal pathway in plants^44^,^45^. To determine whether there was a hypersensitive response (HR) in this process, we assessed the expression pattern of the two HR markers OsHSR203J^46^ and OsHIN1^47^ in the OsAPX8i and control plants that were challenged with PXO99. Both of these marker genes in OsAPX8i plants were induced later than in Os8N3Ri/TP13i plants (Fig. 5a), indicating that there is no fast cell death in the region adjacent to the cuts at the early inoculation stage in OsAPX8i plants. In general, H_2_O_2_ has two opposite actions in that it can lead to either cell wall tightening or loosening depending on the concentration. Thus, the microstructures of these inoculated plants were further analyzed. The transverse sections showed that the adjacent cell walls of the inoculated sites of OsAPX8Ri were loose, whereas those of the OsAPX8-OX line tightened (Fig. 5b). These results indicate that the high concentration of H_2_O_2_ causes the serious dehydration of cells but not the supersensitive reactions.

### OsAPX8 interacts with the susceptibility protein Os8N3/Xa13 in yeast and plants

**Figure 6:**
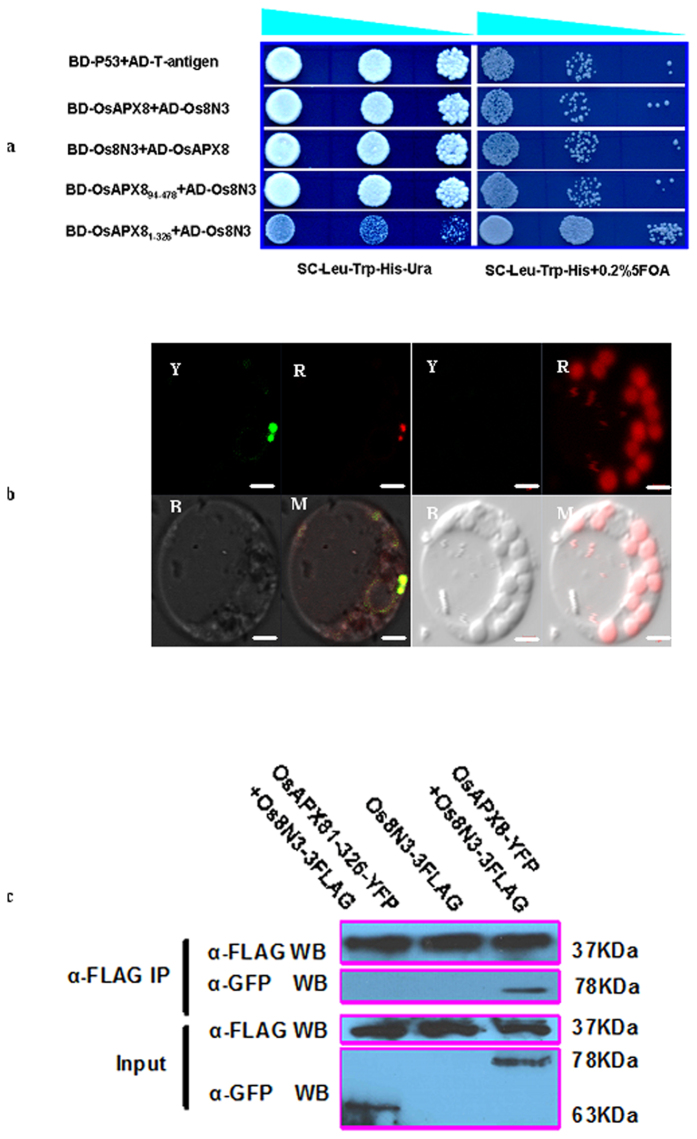
Os8N3/Xa13 specifically interacts with OsAPX8 in yeast and in planta. (**a**) Yeast two-hybrid analysis of the interaction between OsAPX8 and Os8N3/Xa13. The constructs indicated co-transformation into the yeast strain MaV203. Cells grown on the triple-deficient medium (SD, –Trp, –Leu, –His). Cells capable of growing on this medium indicate positive interactions. AD, activation domain of the yeast GAL4 transcription factor; BD, DNA binding domain of the yeast GAL4 transcription factor (the positive control is the reaction of AD-T antigen with BD- p53). (**b**) Confocal micrographs of bimolecular fluorescence complementation (BiFC) in rice protoplasts, showing the interaction of Os8N3/Xa13 with OsAPX8. OsAPX8 proteins expressed as fusions to the C-terminal half of YFP. Os8N3/Xa13 expressed as a fusion to the N-terminal half of YFP. Leaf panel shows the co-expression of OsAPX8-cYFP and Os8N3/Xa13-nYFP plasmids; right panel shows the co-expression of OsAPX8-cYFP and the nYFP empty vector (Y, yellow light; R, red light; B, bright field; M, Merged images). The scale bar is 2 μm. (**c**) OsAPX8 was coimmunoprecipitated with Os8N3. The full-length OsAPX8-YFP or OsAPX81-326-YFP was transfected into transgenic protoplasts with Os8N3-3FLAG overexpression, and the total protein was incubated with an agarose-conjugated anti-FLAG monoclonal antibody. The presence of OsAPX8-YFP and Os8N3-3FLAG in the immune complex was determined by an immune blot.

Because the OsAPX8-OX plants showed a short-term tolerance to *Xoo*, we speculated that some factors were induced during the invasion of the pathogen PXO99 to limit the reaction of OsAPX8. To verify this hypothesis, we carried out a yeast two-hybrid screening. The entire OsAPX8 ORF was fused in frame to the C terminus of the DNA binding domain of GAL4 to create a bait in the plasmid of pDBLeu and was transferred into the yeast strain MaV203 containing three reporter genes, His3, URA3 and LacZ (Invitrogen). OsTFX1, a transcription activator^48^, and murine P53 (CLONTECH, PT3247-1) were used as positive and negative controls, respectively. The bait plasmid pDBLeu-OsAPX8 by itself did not activate the transcription of the reporter genes (Supplementary Fig. S5). The prey library that was constructed from IR24 challenged with PXO99 was screened. In total, 18 yeast clones were obtained as primary positives from the screening of approximately one million yeast transformants. Interestingly, one of these genes encoding OS8N3/Xa13 was screened seven times (Fig. 6a). To confirm the interaction of OsAPX8 with Os8N3/Xa13, we carried out an analysis of bimolecular fluorescence complementation (BiFC). OsAPX8 and Os8N3/Xa13 were fused to the C-terminal and N-terminal parts of yellow fluorescent protein to form the OsAPX8-cYFP and Os8N3/Xa13-nYFP plasmids, respectively and were co-transferred into the TP309 protoplasts. Although there were some perinuclear fluorescent signals occurring, the two co-expressed plasmids produced obvious fluorescent signal in rice chloroplasts; however, no signal was observed when each of these constructs was co-expressed with an empty vector (Fig. 6b). In addition, the interaction between OsAPX8 and Os8N3/Xa13 *in vivo* was also confirmed using coimmunoprecipitation (co-IP) assays. Proteins were extracted from the plastid of rice protoplasts harboring 35S::OsAPX8-YFP and 35S::Os8N3-3× flag constructs and used for co-IP assays. As shown in Fig. 6c, the immunoprecipitation of Os8N3/Xa13 with anti-flag agarose conjugate yielded a co-IP band corresponding to the tagged OsAPX8 that was labeled with the anti-GFP antibody, indicating that the susceptibility protein Os8N3/Xa13 interacts with OsAPX8 even though it was shown to target to the plasma membrane. To assess whether the peroxidase domain or the transmembrane domain interacts with Os8N3/Xa13, we generated two new constructs: BD-OsAPX8_1-326_ (1-326 amino acids) lacking the transmembrane domain and BD-OsAPX8_94-478_ (94-478 amino acids) lacking the low-complexity region. Each of these constructs was then co-transferred into yeast cells with AD-Os8N3/Xa13. The growth of yeast cells containing BD-OsAPX8_94-478_ and AD-Os8N3/Xa13 was noted on selection medium (-Leu-Trp-His-Ura), whereas the cells containing the BD-OsAPX8_1-326_ fusion and AD-Os8N3/Xa13 did not grow. To better characterize the *in vivo* interaction between OsAPX8 and Os8N3 in rice cells, FLAG-tagged Os8N3 and YFP-tagged OsAPX8 and OsAPX8_1-326_ were co-expressed in rice protoplasts. A Co-IP assay showed that Os8N3 interacted with OsAPX8 but that OsAPX8_1-326_ did not (Fig. 6c). These results suggest that the transmembrane domain of OsAPX8 is crucial for the interaction of Os8N3/Xa13 and OsAPX8. This finding is also consistent with the function of Os8N3 as a membrane-anchored sugar transporter^11^. Because chloroplasts are the site of photosynthesis, the produced carbohydrates should be transported to other parts of the cell.

### The functions of OsAPX8 in rice tolerance to salt and bacterial blight can be suppressed by Os8N3/Xa13

To investigate the reaction between Os8N3/Xa13 and OsAPX8 in the salt and bacterial tolerance of rice, we measured the expression patterns of the Os8N3 gene under salt stress and *Xoo* challenge using quantitative RT-PCR (Supplementary Fig. S6). Os8N3/Xa13 was not induced by salt stress or by the *Xoo* race PXO86, but it was induced by the compatible race PXO99 two days after inoculation; however, the induction was later than that of OsAPX8. This result might explain the early tolerance of OsAPX8-OX plants to PXO99 and subsequent overcoming. For the same reason, the OsAPX8-OX plants with no expression of Os8N3/Xa13 showed a durable tolerance to salt stress. Thus, we speculated that Os8N3/Xa13 might suppress the function of OsAPX8 through their interaction.

To verify this hypothesis, we ordered two Tos17 insertion mutants of the OsAPX8 gene from Japan (https://tos.nias.affrc.go.jp). One mutant, NF0576, contained a Tos17 insertion in the 10^th^ exon, whereas the other mutant, NF0595, harbored an insertion in the 11^th^ exon, leading to the destruction of OsAPX8 in the peroxidase and transmembrane domains, respectively (Supplementary Fig. S2). In addition, the mutant oscpk12/NE1534 was also analyzed. This mutant contained a Tos17 insertion in exon 5 of OsCPK12 and had lower expression levels of OsAPx2 and OsAPx8^49^. The resistance phenotypes of these mutants were further checked by inoculation with PXO99. NF0595 showed a durable tolerance to PXO99, whereas NF0576 and Nipponbare did not. The expression of the peroxidase and transmembrane domain of OsAPX8 and Os8N3/Xa13 was further checked by RT-PCR in the plants that were challenged with PXO99 three days after inoculation (3 DAI) (Supplementary Fig. S7a). This result indicates that the peroxidase domain of OsAPX8 is important for rice tolerance to stress and that Os8N3 cannot suppress its function in NF0595. To further explore the role of the OsAPX8 peroxidase domain in tolerance, we constructed the overexpression plasmid of OsAPX8_1-326_ lacking the transmembrane domain, transferred it to the NF0576 mutant harboring a destruction of the peroxidase domain, and obtained 15 hygromycin-positive transgenic plants. All of these overexpression transgenic plants showed durable tolerance to PXO99, and the expression levels of OsAPX8_1-326_ were extremely high (Supplementary Fig. S7a,b). To further examine whether Os8N3/Xa13 suppresses the function of OsAPX8 in rice tolerance to salt, we also generated an Os8N3/Xa13 overexpression construct under the control of the 35S promoter and introduced it into Nipponbare. The resulting transgenic plants were further treated with 300 mM NaCl, alongside the TOS17 mutants. The transgenic NF0576 plant with OsAPX8_1-326_ exhibited higher tolerance to salt than did NF0576, whereas the growth of oscpk12/NE1534, NF0576 and the transgenic plant with the overexpression of Os8N3/Xa13 was significantly suppressed (Supplementary Fig. S7c). In addition, we also checked the tAPX activity in TP309 and Os8N3/X13 transgenic plants that were inoculated with PXO99 and PXO86, respectively. As shown in Supplementary Fig. S8, the activity of OsAPX8 increased in TP13i transgenic plants, whereas it was suppressed in TP13 overexpression plants; this activity peaked on the third day and then decreased in TP309 when challenged with PXO99 (Supplementary Fig. S8a). However, this activity increased in TP309 when challenged with PXO86 (Supplementary Fig. S8b). These results indicate that OsAPX8 activity is negatively correlated with the expression of Os8N3/Xa13. Taken together, these results show that OsAPX8 increases rice tolerance to biotic and abiotic stress, which can be suppressed by Os8N3/Xa13 through the interaction of their transmembrane domains.

## Discussion

Most ascorbate peroxidases are involved in plant development and abiotic stress^35^,^37^,^38^,^39^,^41^,^50^. However, the response of these peroxidases to pathogen signal has rarely been reported. Only OsAPX1 and OsAPX2 have been shown to respond to blast pathogen (*Magnaporthe grisea*)^50^. In this study, OsAPX2, OsAPX4, OsAPX6, OsAPX7 and OsAPX8 were shown to respond to the bacterial pathogen *Xoo*. The high response to both PXO86 and PXO99 indicates that OsAPX8 may be involved in broad-spectrum resistance or plant innate immunity to bacterial blight. To further understand the function of OsAPX8 in broad-spectrum resistance, we produced transgenic plants with the overexpression and RNAi suppression of rice tAPX (OsAPX8). These RNAi suppression transgenic plants were hypersensitive to high salt or *Xoo* but showed a normal morphological phenotype and did not exhibit symptoms of oxidation when grown under normal or lower-salt conditions. The OsAPX8-OX transgenic plants showed a tolerance to higher salt and *Xoo*, although this tolerance could be overcome by Os8N3/Xa13. These results indicate that the function of tAPX in H_2_O_2_ scavenging in OsAPX8Ri transgenic plants can be replaced by the other ascorbate peroxidases when grown under low-salt or normal conditions but not under high-salt conditions or *Xoo* infection. Thus, OsAPX8 is the major H_2_O_2_ scavenging enzyme when rice is challenged with a high salt concentration or with the *Xoo* strains PXO99 or PXO86.

Because OsAPX8 targets to chloroplasts, it should interact with some protein in chloroplasts. However, our results showed that OsAPX8 has a strong interaction with the plasma membrane protein Os8N3/Xa13. Os8N3/Xa13 was previously shown to target to the plasma membrane through observation of tobacco leaf epidermal cells^8^. We repeated two protein interaction experiments several times using a yeast two-hybrid system. OsAPX8 certainly interacts with Os8N3/Xa13; thus, we examined the interaction between Os8N3/Xa13 and OsAPX8 *in vivo*. The results of both BiFC and co-IP assays showed that this interaction mainly occurs in chloroplasts. In addition, there were also some perinuclear fluorescent signals occuring in the TP309 protoplasts with the two co-expressed plasmids, indicating that the fluorescent signals from the various autofluorescent and light-scattering components of cells can result in background noise or the interaction between Os8N3/Xa13 and OsAPX8 may exist in perinuclear region or cytoplasm in addition to chloroplasts. This requires to be further examined through precision instrument in the future. Although this result seems paradoxical, it is not impossible. First, a protein structural analysis using the ChloroP1.1 program (http://www.cbs.dtu.dk/services/ChloroP/) and WoLF PSORT (http://www.genscript.com/results/143012805716365.html) predicted that Os8N3/Xa13 has the characteristics of chloroplast proteins (Supplementary Table 3). Second, Os8N3/Xa13 may target the chloroplast inner membrane from the origin of the chloroplast. In theory, the outer membrane of the chloroplast is derived from the ER, whereas the inner membrane originated from the cell membrane of a cyanobacterium^51^. In addition, the thylakoid lipid bilayer shares characteristic features with prokaryotic membranes and the inner chloroplast membrane^52^. Third, Os8N3 in chloroplasts is not as abundant as in the plasma membrane and could not be easily detected using existing technical equipment in a previous study^8^. The interaction of OsAPX8 with Os8N3/Xa13 was observed, although more evidence is required to elucidate this complex and elaborate life process.

Bacterial blight is generally distinguished into four types, including lesions found at the margin of leaf blades forming a wavy infection and yellowing pattern and the “kresek” symptom, with serious cell dehydration and the leaves turning grayish-green, rolling up and withering. In this study, the overexpression of OsAPX8 delayed cell dehydration and wilting and the replication of *Xoo* during the infections, and no hypersensitivity took place. In contrast, the plants of OsAPX-RNAi showed rapid cell dehydration and wilting but no hypersensitivity under the same treatment, although their H_2_O_2_ content was extremely high (Fig. 4c). These results indicate that H_2_O_2_ does not induce the hypersensitive response but is the cause of cell dehydration. This conclusion was further confirmed by the expression of the hypersensitive response (HR) marker genes OsHSR203J and OsHIN1. These genes were induced considerably later, indicating that the excess H_2_O_2_ in these plants is not the cause of HR (Fig. 5a). In addition, the content of H_2_O_2_ can influence the structure of the cell wall. An anatomical analysis of inoculated leaves showed that the cell walls of the OsAPX8 overexpression plant were more compact than were those of TP309 with empty vector, whereas those of the OsAPX8 RNAi plant were the loosest. In general, our results agree with those of previous studies^53^,^54^,^55^. Thus, a high concentration of H_2_O_2_ might be the main cause of the “kresek” symptom of bacterial blight in rice without major resistance genes.

Pathogens face three barriers when infecting cells. Once the first barrier, the cell wall, is breached, the pathogens are recognized by plant membrane pattern recognition receptors (PRRs) and activate pathogen- or microbial-associated molecular pattern (PAMP or MAMP, respectively)-triggered immunity (PTI or MTI)^56^. To further inhibit PTI, pathogens can secrete virulence effectors into plant cells to active or suppress the expression of some plant genes. Effector-triggered immunity (ETI), the third barrier, will be activated if the virulence effector is recognized by a cognate disease-resistant gene. H_2_O_2_ has been previously recognized as an integral aspect of plant immunity and is considered unspecific because it can be induced by all types of pathogens and other non-biotic stresses, such as wounding, salt and chilling injury. H_2_O_2_ not only causes the structure of the cell wall to become more compact at low levels^54^ but can also act as a signal molecular to activate redox-sensitive transcription factors, such as HSF, WRKY, RAV, Myb and NPR1, to trigger basal immunity, including systemic acquired resistance (SAR)^57^. Here, OsAPX8 overexpression was shown to confer transgenic plants a strong disease tolerance to *Xoo* during early infection and lead to a compacted cell wall, as previously reported. Hence, we believe that OsAPX8 is primarily involved in basal defense through the scavenging of excess H_2_O_2_ in chloroplasts during the early infection of *Xoo*.

**Figure 7:**
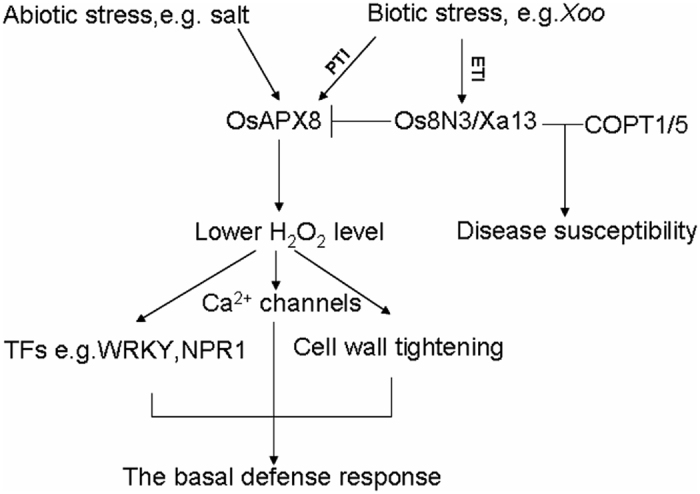
Model for the structure and regulation of the basal defense or tolerance response mediated by OsAPX8 through PTI (pathogen-associated molecular pattern triggered immunity) pathway. Os8N3/Xa13 is induced in the chloroplast inner membrane by PXO99 through the ETI (effector-triggered immunity) pathway, where it interacts with tAPX to reduce its ability to detoxify the H_2_O_2_. The arrows indicate the positive regulation of the pathway, whereas blunted lines indicate the negative regulation of the pathway.

Os8N3/Xa13 is a member of the MtN3/saliva/SWEET gene family and is induced by the effector pthxo1, which has previously been considered a host gene for strain-specific susceptibility to the bacterial blight disease of rice^8^,^11^,^12^. In fact, MtN3/saliva/SWEET-type genes are rather conservative and are involved in multiple physiological processes, including reproductive development, senescence, environmental adaptation, and host–pathogen interactions in higher-order plants. Thus, these genes are ideal copartners for overcoming the second or third defensive line by virulence effectors in plant cells after long-term selection. Fig. 7 shows the model for the structure and regulation of the basal defense or tolerance response as mediated by tAPX. For example, Xa13/Os8N3 is induced by PXO99 to cooperate with COPT1 and COPT5 to promote the removal of copper from xylem vessels, which helps *Xoo* to multiply and spread, finally causing the disease^18^. Here, our results indicate that Os8N3/Xa13 interacts strongly with the chloroplast ascorbate peroxidase OsAPX8 to suppress the basal defense to PXO99, providing new confirmation for this hypothesis. Namely, the induction of Os8N3/Xa13 by PXO99 is likely the main cause of the short tolerance to *Xoo* in plants with the overexpression of OsAPX8.

## Methods

### Plant material and growth conditions

The rice plant (*O. sativa*) cv. TP309 was used in this study. Plants were grown in a greenhouse and field. The T1 generation of overexpression, RNAi and control plants that were transformed through *Agrobacterium tumefaciens* (strain EHA105)^58^ were treated with salt and *Xoo*.

### Plasmid construction and transformation of rice

The complete and partial coding regions of OsAPX8 (AB114856) and Xa13/Os8N3 were amplified from rice total RNA using RT-PCR. The following primers pairs were used: 5′-AGAATTCGGTACCatggcggagcgcatcgcc-3′ and 5′-ATCTAGACTAGTCGACgctcccgagcagagacgtc-3′ or 5′-ATCTAGACTAGTCGACATCCTCTGCATATTTTTCGGC-3′ for the overexpression and subcellular location of OsAPX8 or OsAPX8_1-326_; 5′-CCAGCCGTCGAAGAGAAGGATCC-3′ and 5′-CACATGCCCATCCTTTTACCTC-3′ for OsAPX8RNAi; 5′-GTCGACCatggcggagcgcatcgcc-3′ and 5′-actagtGCTCCCGAGCAGAGACGTC-3′ or 5′-actagtATCCTCTGCATATTTTTCGGC-3′ for pDBLeu-OsAPX8 or pDBLeu Leu- OsAPX8_1-326_; 5′-GTCGACCGGCGGCCTCCGCCTCCGCTCC-3′ and actagtGCTCCCGAGCAGAGACGTC-3′ for pDBLeu-OsAPX8_94-478_; and 5′-GGAATTCGGTACCGACTACAAAGACGATGACGACAAGGACTACAAAGA CGATGACGACAAGGACTACAAAGACGATGACGACAAGATGGCAGGAGGTTTCTTGTCCATGG-3′ and 5′-AACTAGTGGATCCCACGGCGGCGGTGATCTCG-3′ for 3× FLAG-Xa13/Os8N13-OX. the PCR products for RNAi construction were cloned into the Gateway vector (pANDA)^12^, in which hairpin RNA is driven by maize ubiquitin promoter and an intron 5′ upstream of inverted repeats. Agrobacterium-mediated transformation was performed as previously described.

### Determination of tolerance to salt stress

For germination tests, rice seeds were surface sterilized for 5 min with ethanol (75% v/v) and 10 min with commercially diluted (1:3 v/v) NaOCl twice, followed by 3 to 5 rinses with sterile distilled water. Germination was carried out for 72 h on sterile half-strength Murashige and Skoog (MS) medium containing 200 mM, 250 mM or 300 mM NaCl in the dark at 28 °C day/25 °C night temperatures, 12 h light/12 h dark cycle, and 50% humidity.

For the salt stress treatments, 1-week-old seedlings of wild-type, TP309 and OsAPX8 transgenic rice were cultured on ½ MS medium supplemented with 100 mM, 250 mM, or 300 mM NaCl for seedling stage tests. Wild-type and transgenic plants that were grown in soil were used to determine the stress tolerance of adult plants at different times, including the tillering, elongation-heading and mature stages.

### Yeast two-hybrid experiment

Yeast two-hybrid assays were performed in accordance with the ProQuest Two-Hybrid System Manual (Invitrogen). OsTFX1^48^ and Os8N3^12^ were used as the positive and negative controls, respectively, for the transcription activation analysis of OsAPX8. The transformation efficiency was estimated by plating an aliquot of transformation mixture onto SD/-Leu-Trp medium. Candidates that were selected on SD/-Leu-Trp-His medium were subjected to uracil and 5FOA assays according to the manufacturer’s instructions (Invitrogen).

### Co-IP analysis

Rice protoplasts were transformed with 3ХFLAG-Xa13/Os8N13-OX alone or in combination with OsAPX8-YFP or OsAPX8_1-326_-YFP. The total protein was immunoprecipitated with anti-FLAG antibody, and the presence of FLAG-tagged proteins and OsAPX8-YFP in the same immunocomplex were assessed with monoclonal anti-FLAG antibody (Sigma-Aldrich) and anti-GFP antibody (Abcam), respectively.

### APX activities

Fresh leaves (100–200 mg fresh weight) were frozen in liquid N_2_, ground, and mixed with 2 mL of a 50 mM MES/KOH buffer (pH 7.0) containing 40 mM KCl, 2 mM CaCl_2_, 1% (w/v) poly-vinyl-pyrrolidone, and 1 mM AA. After thawing, 0.1% (v/v) Triton X-100 was added to the mixture, and the tubes were gently mixed for 15 min at 4 °C. Homogenates were then centrifuged at 4,500× *g* for 2 min, and the supernatants were used to measure the foliar APX activity. For thylakoid isolation, leaves were homogenized with grinding solution (0.5 M sucrose, 25 mM HEPES, and 2 mM EDTA, pH 7.6) and passed through four layers of cheesecloth to remove debris. The pre-clearing homogenates were centrifuged at 3,000× *g* for 10 min, and the chloroplasts pellet was further washed with grinding solution and resuspended in extracting buffer (5 mM AA, 1 mM EDTA, and 100 mM potassium phosphate buffer, pH 7.0). APX was measured spectrophotometrically by a modified version of the method of Nakano and Asada^59^. The reaction mixture (950 μL) contained 50 mM KH_2_PO_4_/K_2_HPO_4_ buffer (pH 7.0), 500 μM AA, and 0.1 mM H_2_O_2_. A 50 μL volume of foliar homogenates or chloroplasts proteins was added to the reaction mixture and gently mixed. The oxidation of AA was followed by a decrease in A_290_ in a spectrophotometer at 25 °C. The reaction rates were linear for at least 3 min and were corrected for AA auto-oxidation in the presence of 0.1 mM H_2_O_2_. The APX activity was calculated using an extinction coefficient of 2.8 mM^−1^ cm^−1^ for AA^27^. The chloroplasts proteins were also loaded onto a native gel to detect the tAPX activity. Running gel (7%): 6.8 mL of 30% acrylamide mix, 22.4 mL of 1 mol/L Tris-HCl (pH 8.8), 0.8 mL of 1% APS, and 40 μl TEMED. Stacking gel (4%): 2.6 mL of 30% acrylamide mix, 2.6 mL of 1 mol/L Tris-HCl (pH 6.8), 0.6 mL of 1% APS, and 30 μl of TEMED. Running buffer: 25 mM Tris-HCl (pH 8.3), 192 mM glycine, and 1 mM ASA. Electrophoresis was performed for 2 h at a constant 100 V. After electrophoresis, the gel was washed 3 times in 10 mL ddH_2_O containing 1 mM ASA and then incubated for 1–2 min in following the staining solution: 10 mg TMB (3,3′,5,5′-Tetramethylbenzidine) (T2885–100 mg, Sigma) in 2.5 mL ethanol, 50 mL acetate acid/sodium acetate buffer solution (pH 4.5), 3 mL of 3% H_2_O_2,_ 47.5 mL of ddH_2_O and a small amount of ASA^53^,^60^.

### H_2_O_2_ content assay

Hydrogen peroxide was measured using the Ferric Xylenol Orange method as described previously^61^. Briefly, leaf tissue was homogenized in cold acetone and filtered to remove cellular debris. The supernatants were extracted with CCl_4_-CHCl_3_. Then, the extract was added to a tube containing 250 μM ferrous ammonium sulfate, 100 μM sorbitol, and 100 μM xylenol orange in 25 mM H_2_SO_4_. After 30 min in the dark at room temperature, the absorbance was read at 560 nm.

### Subcellular localization and BiFC assays

To visualize the subcellular localization of OsAPX8, the 1435-bp full-length coding sequence (CDS) of OsAPX8 without a stop codon was cloned into the 35S::YFP vector driven by the 35S promoter with YFP in the frame at the C terminus. Protoplasts isolated from 2-week-old TP309 plants were transfected with the YFP constructs as described^62^. The YFP and RFP fluorescence were examined under a confocal microscope (Zeiss LSM 710 NLO) at excitation wavelengths of 514 and 561 nm, respectively. For the BiFC assay, the cYFP-OsAPX8 and nYFP-Os8N3 plasmids were coexpressed in rice protoplast.

### RT-PCR

The total RNA from leaves was isolated using Trizol (Invitrogen). First-strand cDNA was synthesized from 2 μg of total RNA using MMLV reverse transcriptase (Promega) and oligo (dT)_15_ primer. RT-PCR and qPCR were performed as described previously^6^,^63^. The RT-qPCR primer pairs for the OsAPX1 to OsAPX8 genes that were used here were described previously^35^. All of the RT-qPCR primers are listed in Supplementary Table S2.

### Electrolyte leakage test

The relative electrolyte leakage was determined according to a previous description^64^. The rice leaves that were inoculated with PXO99 were used to evaluate the electrolyte leakage by determining their relative conductivity in solution. Each data point represents the average of three independent experiments. The data were subjected to statistical analysis using a t test.

### Bacterial blight assays

Bacterial inoculations were performed using the leaf clip inoculation of transgenic plants; each line was treated for 10 plants with 10^5^ cells/mL of *Xoo* strain PXO99 as the initial concentration. Bacterial colony-forming units (cfu) were estimated as described for a receptor kinase-like protein encoded by the rice disease resistance gene.

## Additional Information

**How to cite this article**: Jiang, G. *et al*. The rice thylakoid membrane-bound ascorbate peroxidase OsAPX8 functions in tolerance to bacterial blight. *Sci. Rep.* **6**, 26104; doi: 10.1038/srep26104 (2016).

## Supplementary Material

Supplementary Information
