# Supplementary material for: The rice thylakoid membrane-bound ascorbate peroxidase OsAPX8 functions in tolerance to bacterial blight
Source: Sci Rep. 2016 May 17;6:26104. doi: 10.1038/srep26104 (PMC4868969; doi:10.1038/srep26104)
Supplement: Supplementary Information [file srep26104-s1.doc]

#### The rice thylakoid membrane-bound ascorbate peroxidase OsAPX8 functions on tolerance to bacterial blight

#### Guanghuai Jiang, Dedong Yin, Jiying Zhao, Honglin Chen^†^, Lequn Guo, Lihuang Zhu^*^ and Wenxue Zhai^*^

Supplementary Information accompanies this paper at <http://www.nature.com/srep>


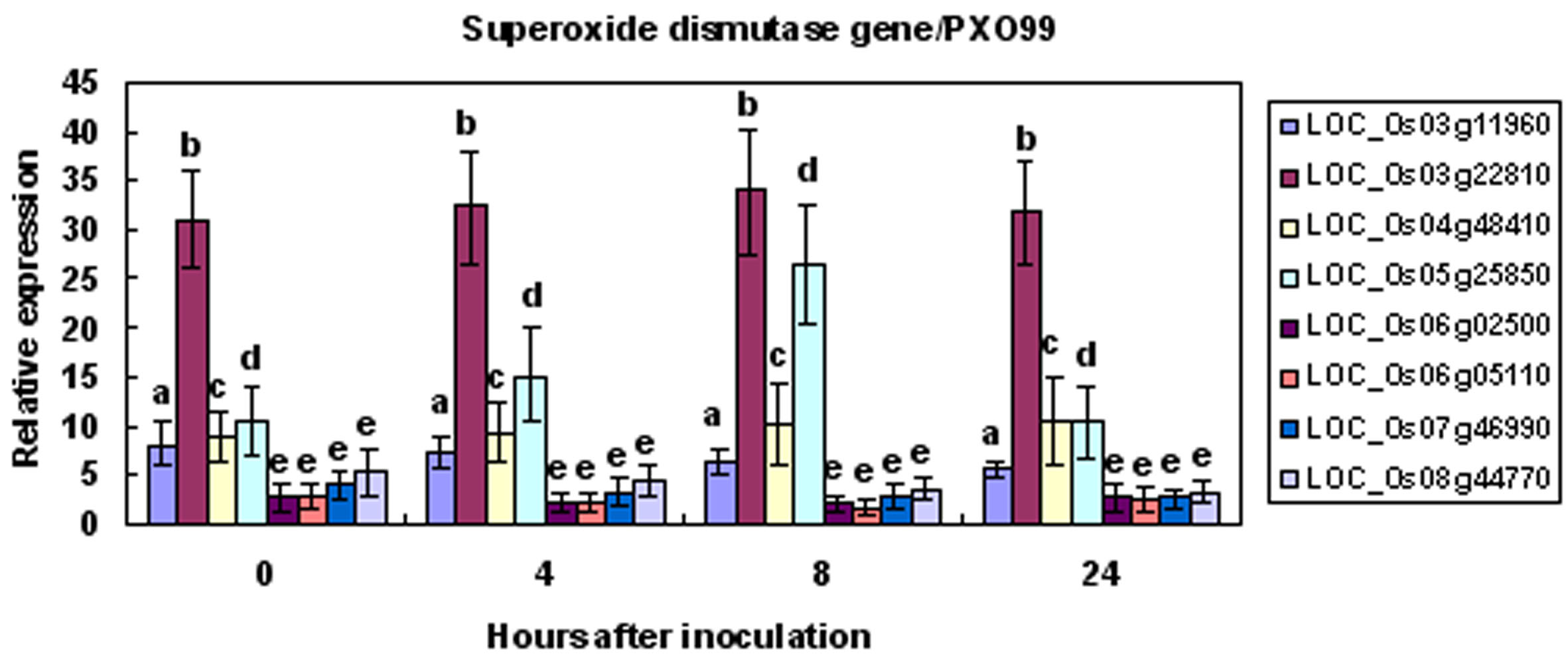

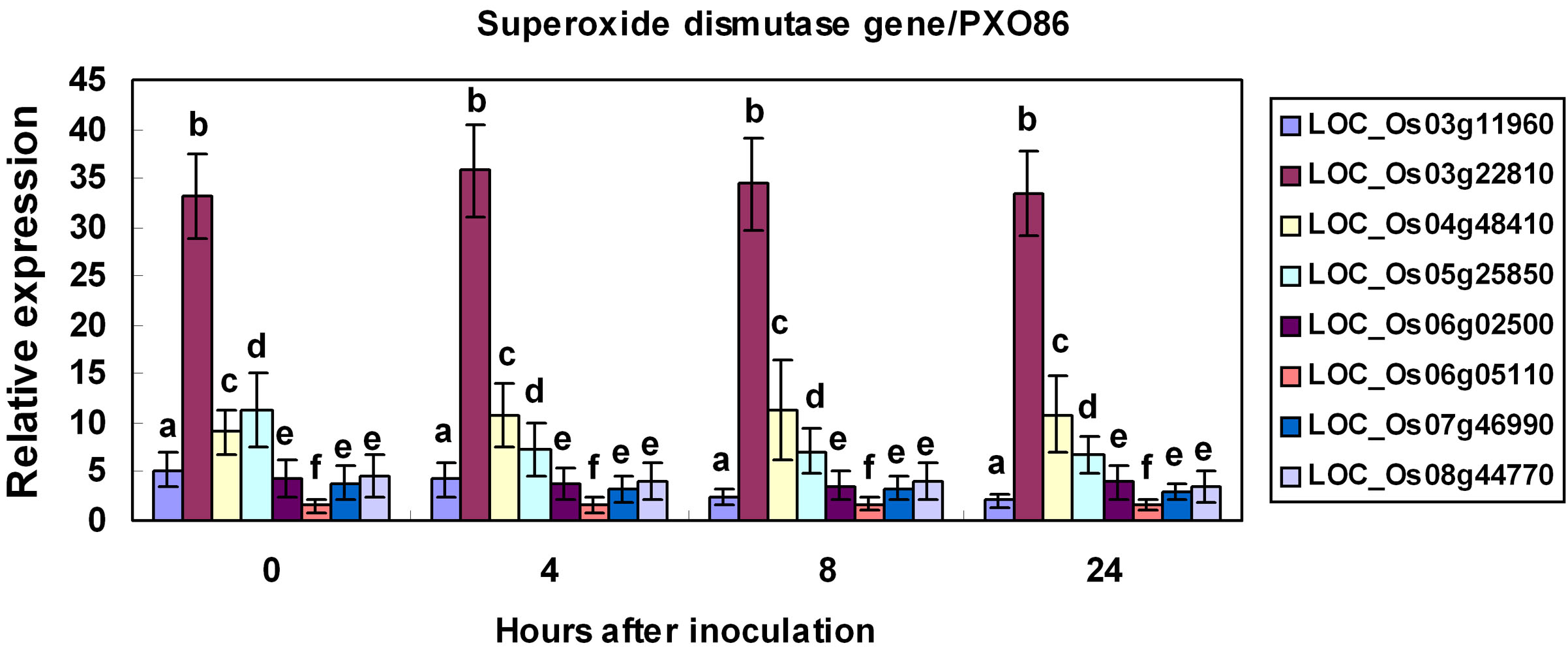


A


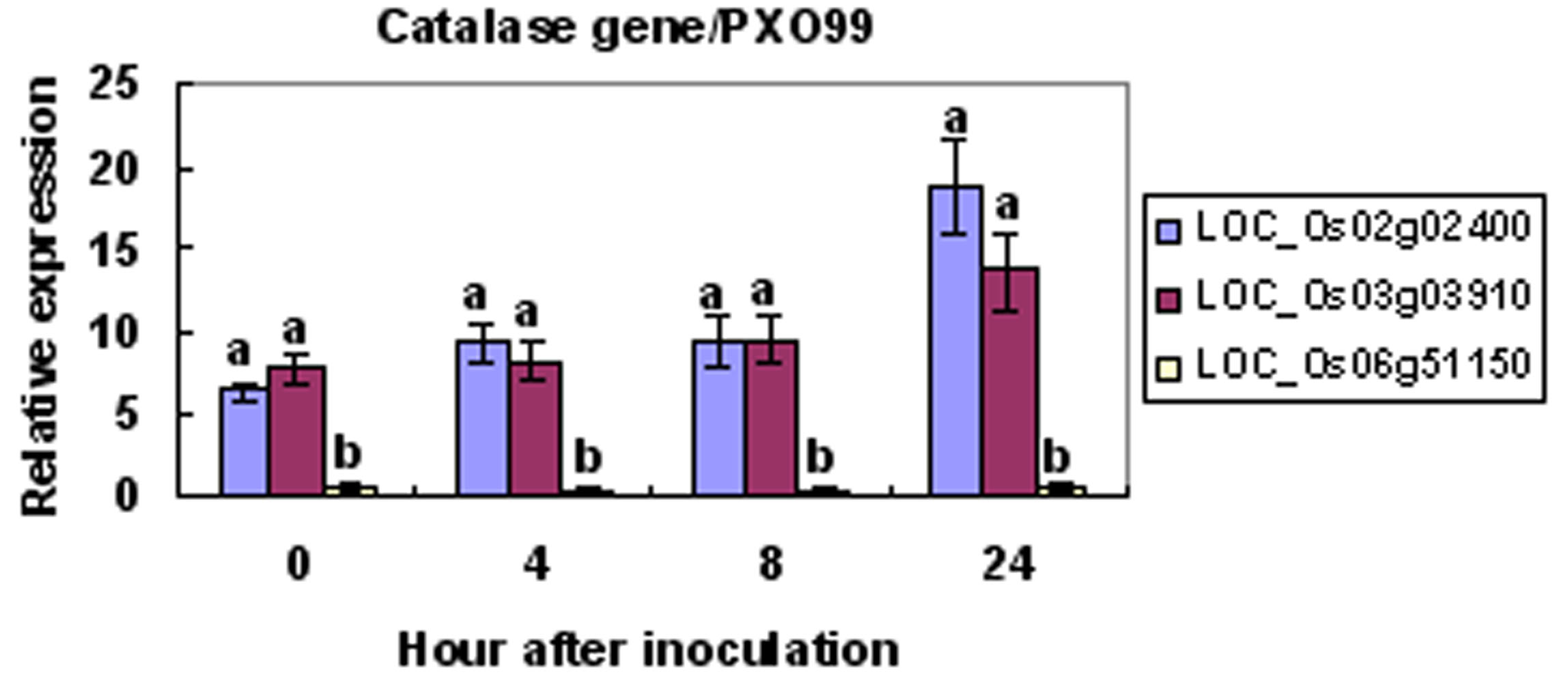

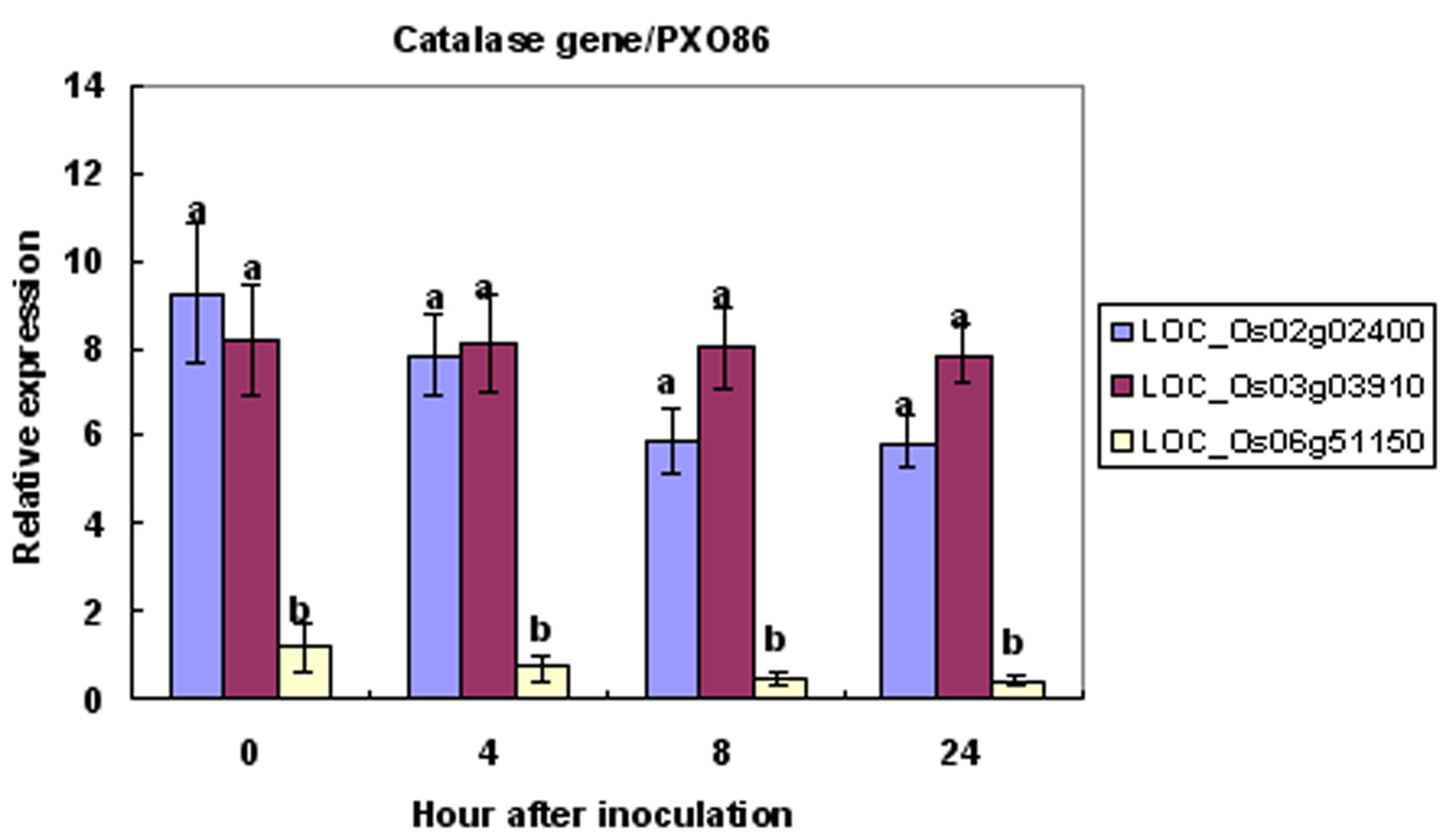


B


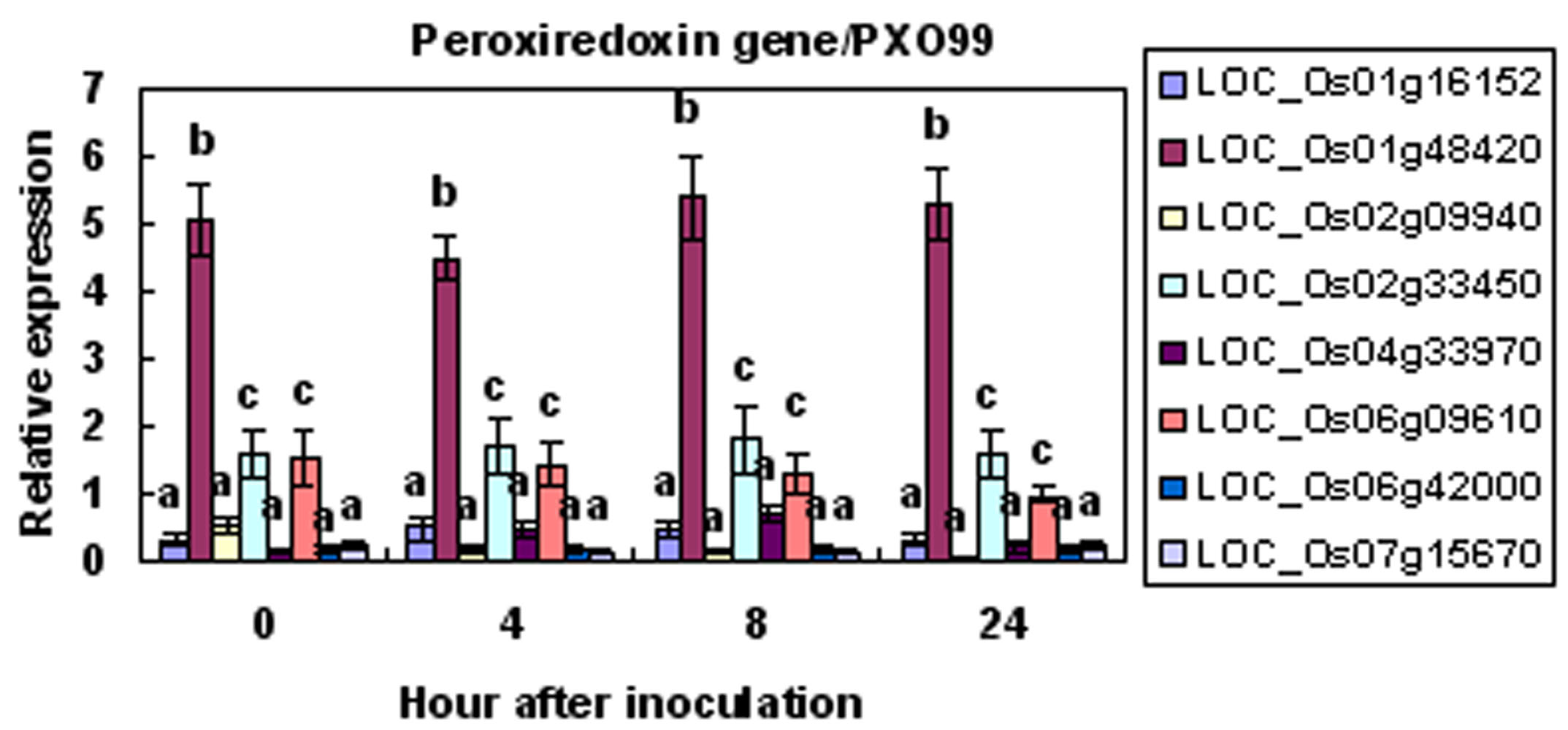

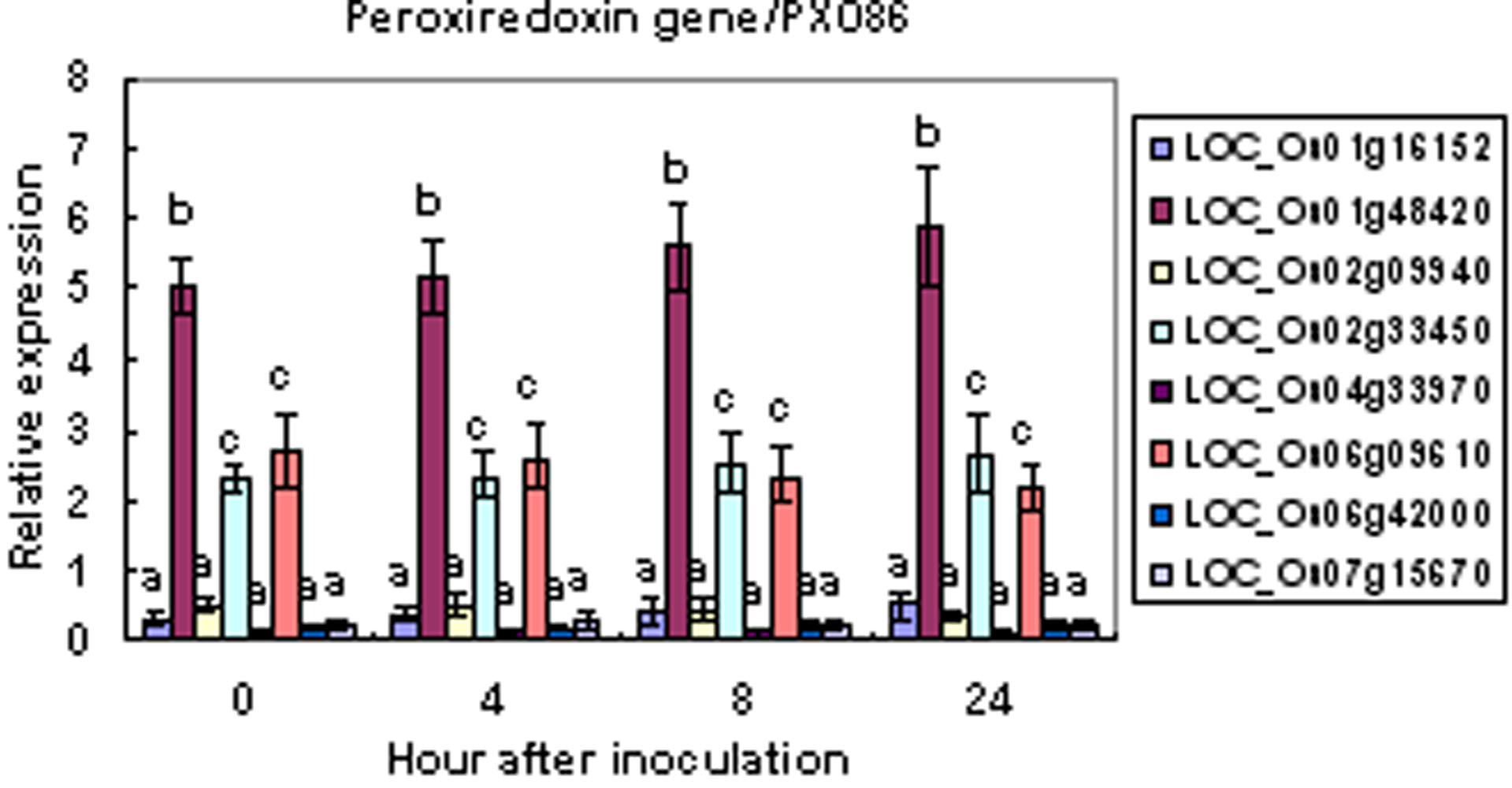


C


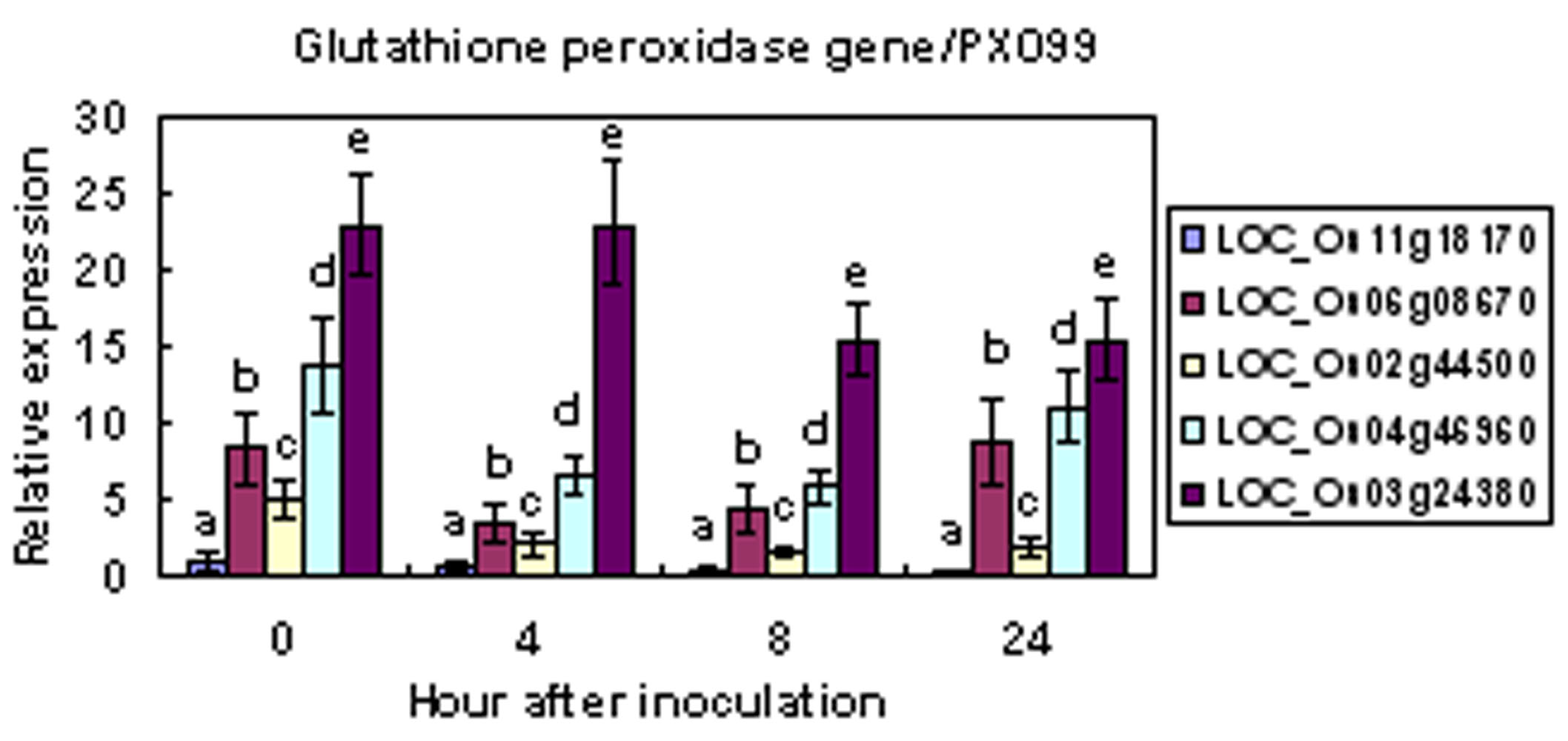

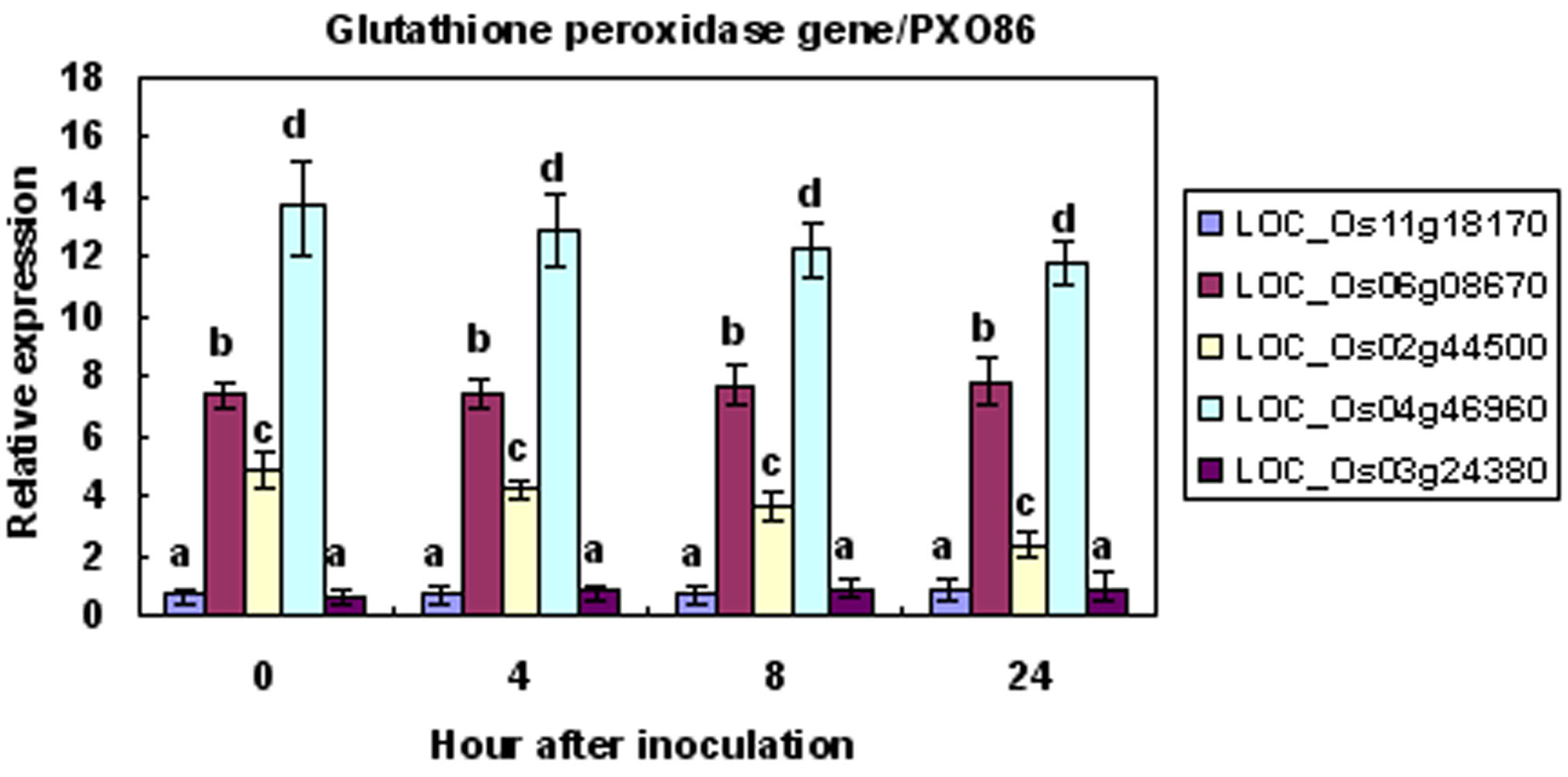


D

Supplementary Figure 1. Expression of the rice catalase, peroxiredoxin and glutathione peroxidase genes in leaves. (a) Quantitative RT-PCR analysis of 8 superoxide dismutase mRNAs that accumulated in TP309 plants. (b) Quantitative RT-PCR analysis of 3 catalase mRNAs that accumulated in TP309 plants. (c) Quantitative RT-PCR analysis of 8 peroxiredoxin mRNAs that accumulated in TP309 plants. (d) Quantitative RT-PCR analysis of 5 glutathione peroxidase mRNAs that accumulated in TP309 plants. Leaves that were inoculated with *Xoo* strain PXO99 or PXO86 were harvested at 0, 4, 8, and 24 h. The values represent the means ±SD of three replicates.


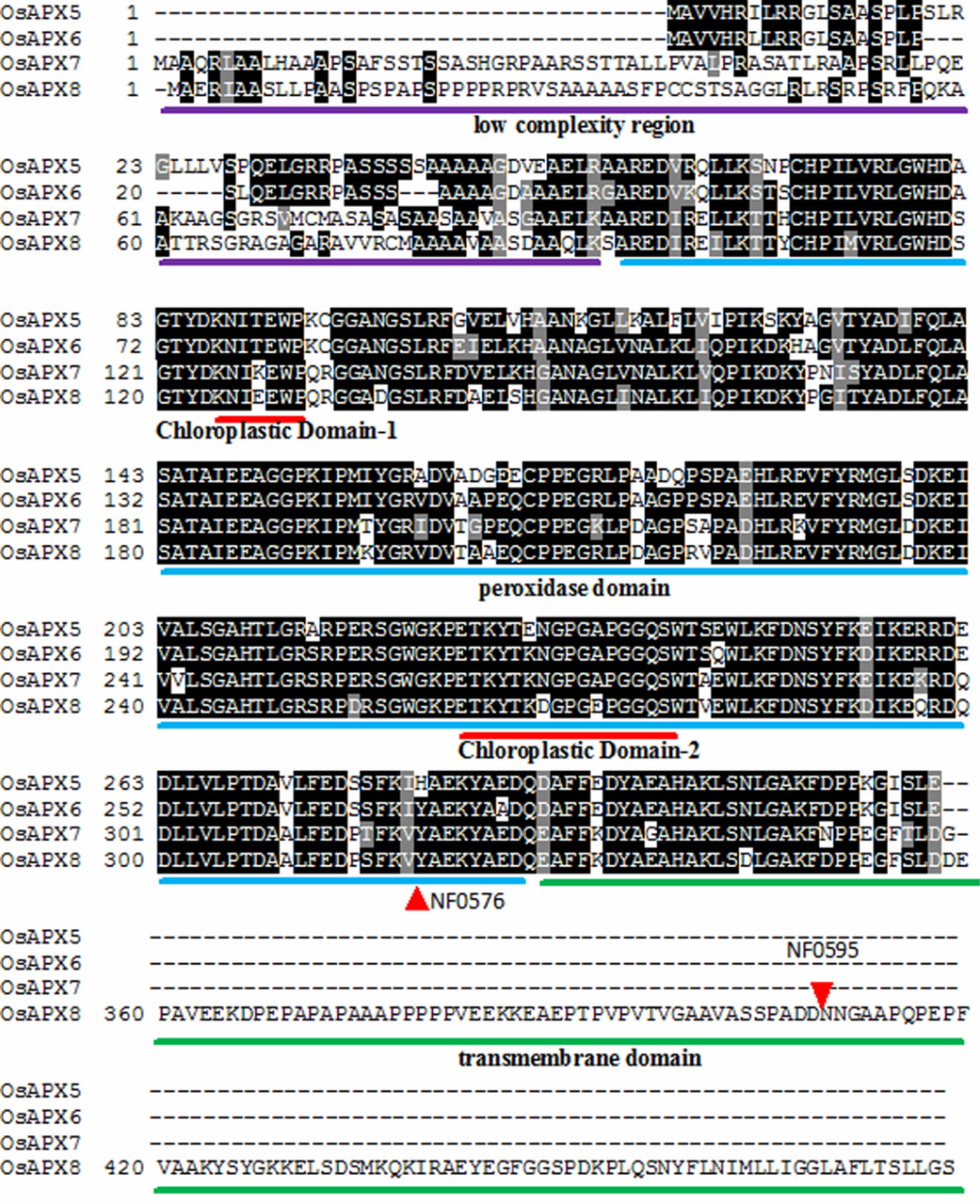


Supplementary Figure 2. Sequence alignments of rice chloroplastic APXs. The chloroplastic domains 1 and 2 are shown (read line area). The purple and blue line areas are a low-complexity region and peroxidase domain, respectively. The sense primer for the construction of OsAPX8RNAi is located in the beginning of the thylakoid-bound transmembrane domain (green line area); the reverse primer is located in the 3’-UTR (not shown here).


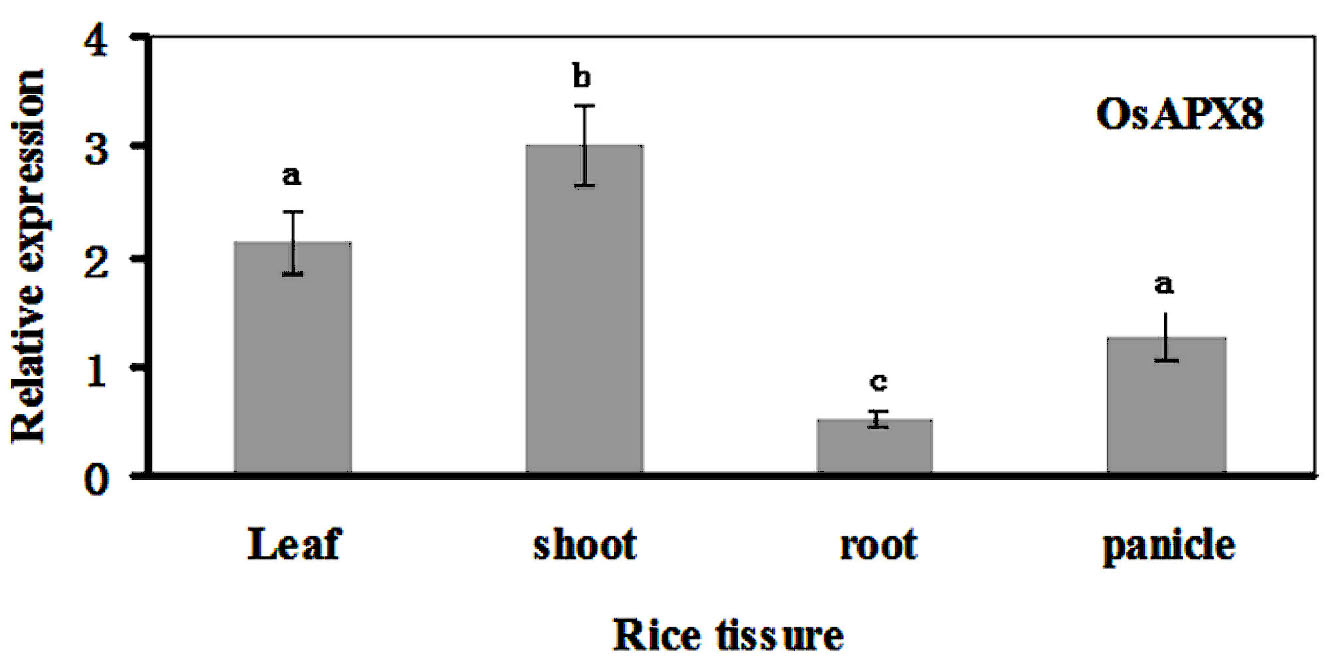


Supplementary Figure 3. Quantitative RT-PCR analysis of *OsAPX8* expression in various tissues of TP309. The expression level of an actin gene was used as an internal control. All of the data are presented as the means ± S.D. (n=3). Different letters (a-c) indicate significant differences (P<0.05) between lines.


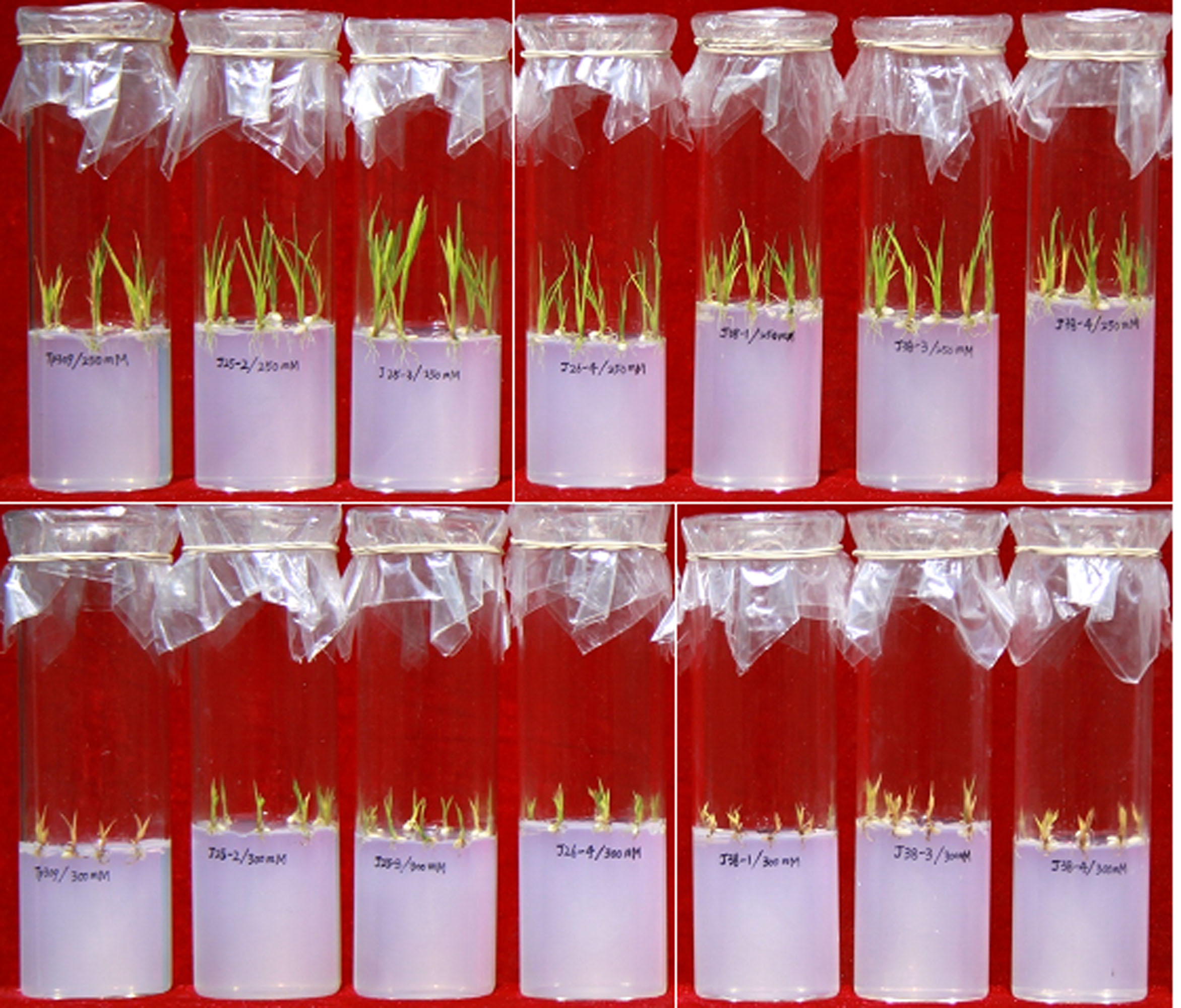


Supplementary Figure 4. Analysis of salt tolerance in *OsAPX8* transgenic rice. J25-2, J25-3 and J26-4 are OsAPX8 overexpression lines; J38-1, J38-3 and J38-4 are OsAPX8 RNAi lines (upper panel – plants that were treated with 250 mM NaCl; lower panel – plants that were treated with 300 mM NaCl).


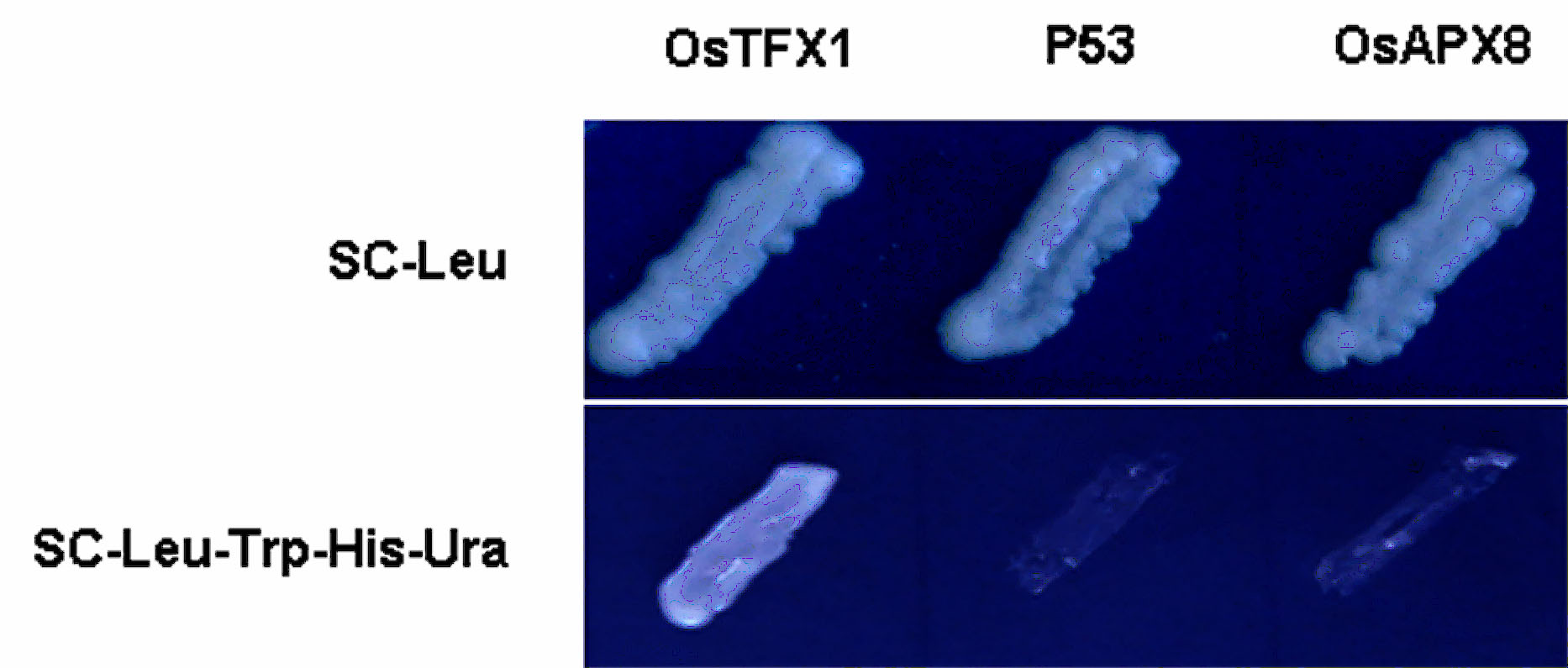


Supplementary Figure 5. OsAPX8 did not activate the transcription of reporter genes by itself in yeast. OsTFX1 and P53 were used as positive and negative controls, respectively.


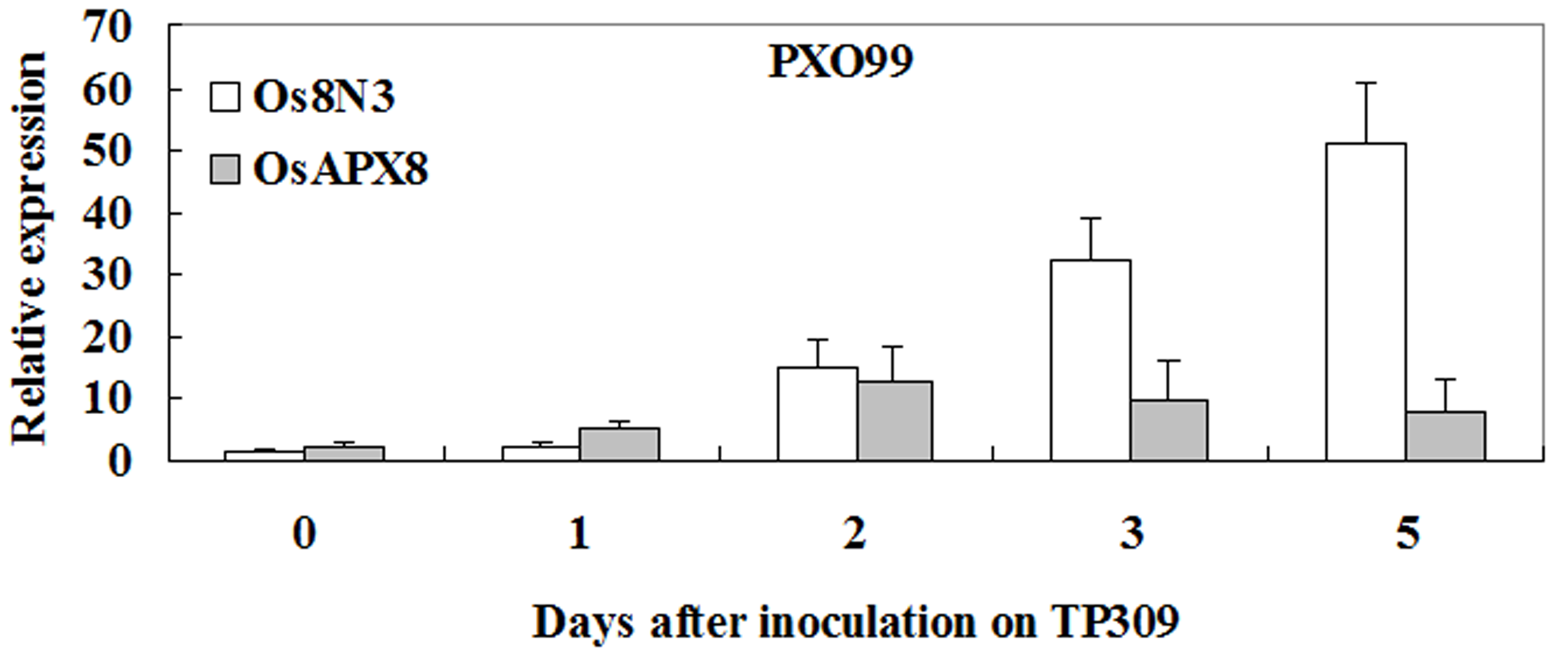


A


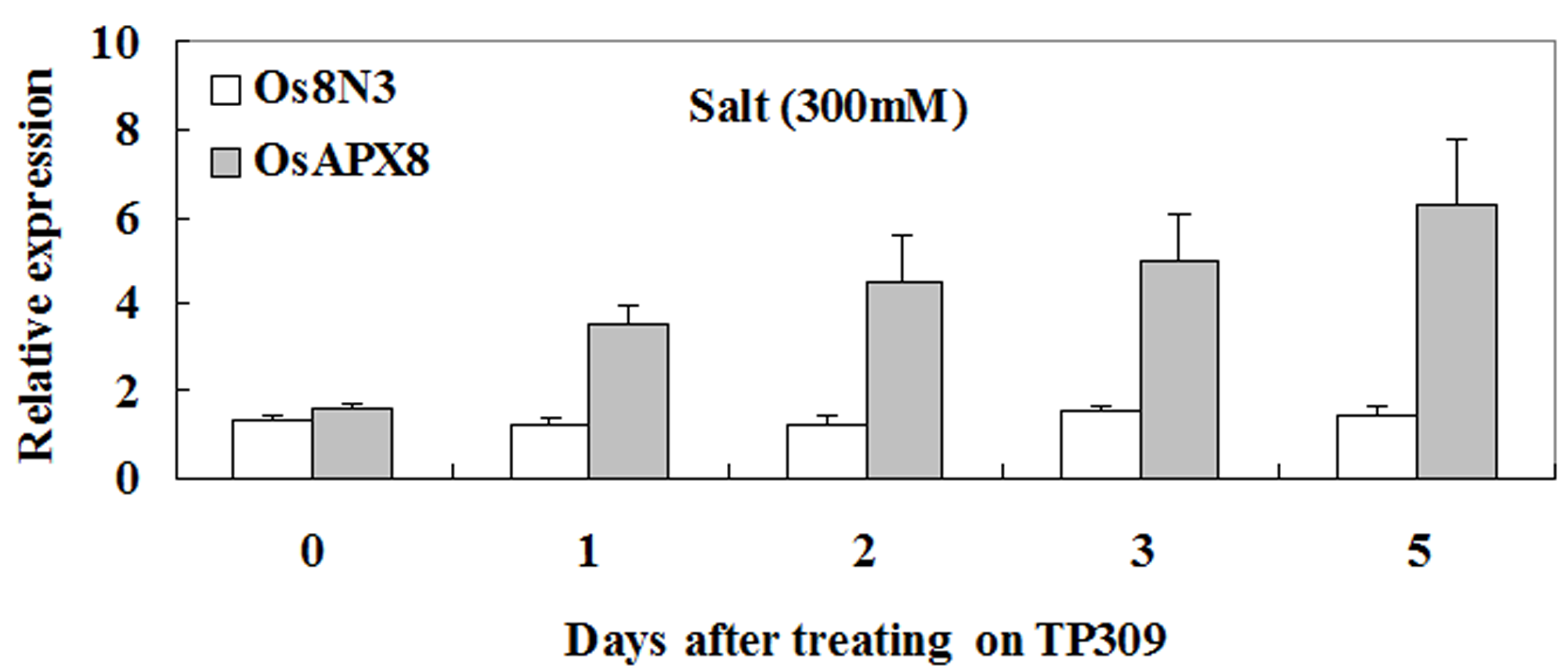


B


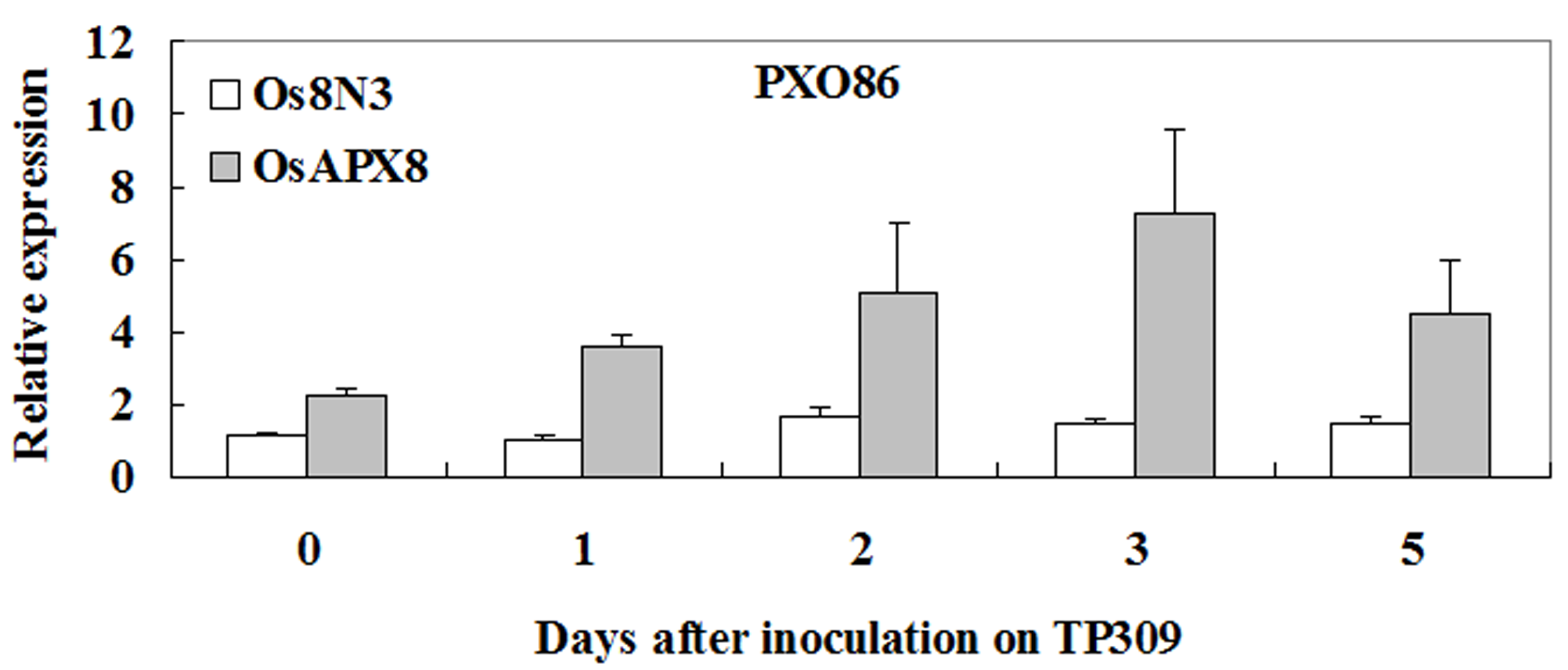


C

Supplementary Figure 6. Quantitative RT-PCR analysis of Os8N3 and OsAPX8 under stress. (a) TP309 was inoculated with *Xoo* strain PXO99 (OD600 was 0.5). The induced expressions of Os8N3 and OsAPX8 was checked 1, 2, 3 and 5 days after inoculation. (b) The expression of Os8N3 and OsAPX8 in the leaves of TP309 treated with NaCl (300 mM). (c) The expression of Os8N3 and OsAPX8 in leaves of TP309 treated with *Xoo* strain PXO86 (OD600 is 0.5). The data represent means ±S.D. for at least three independent experiments.


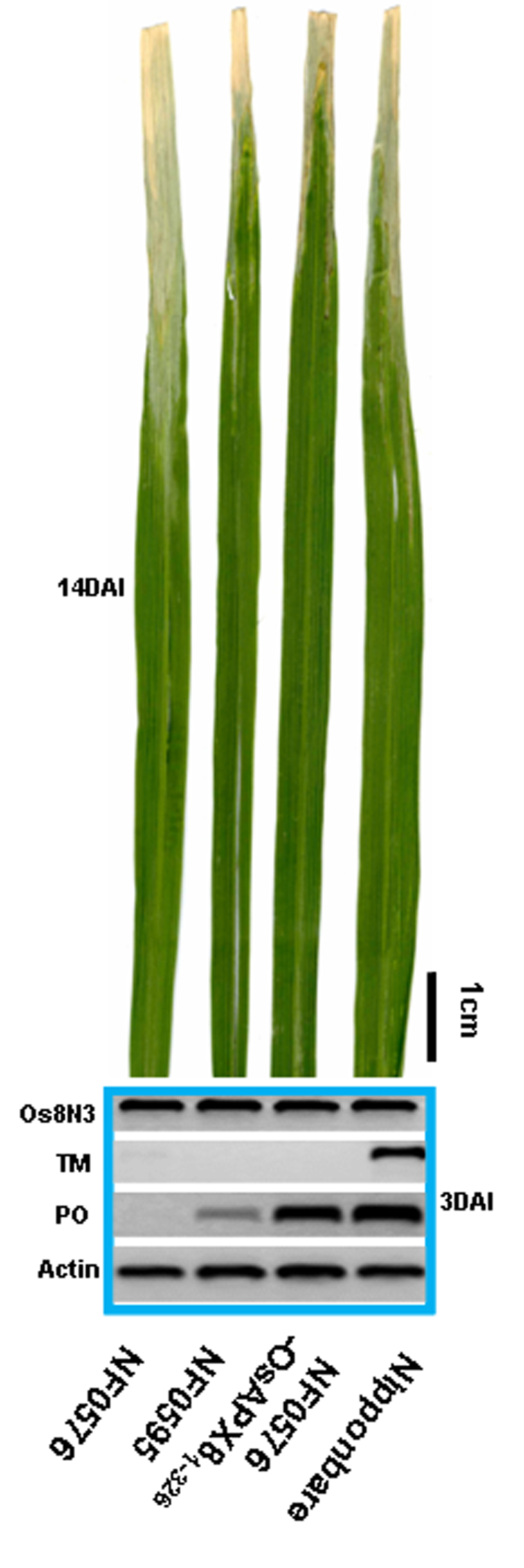


B


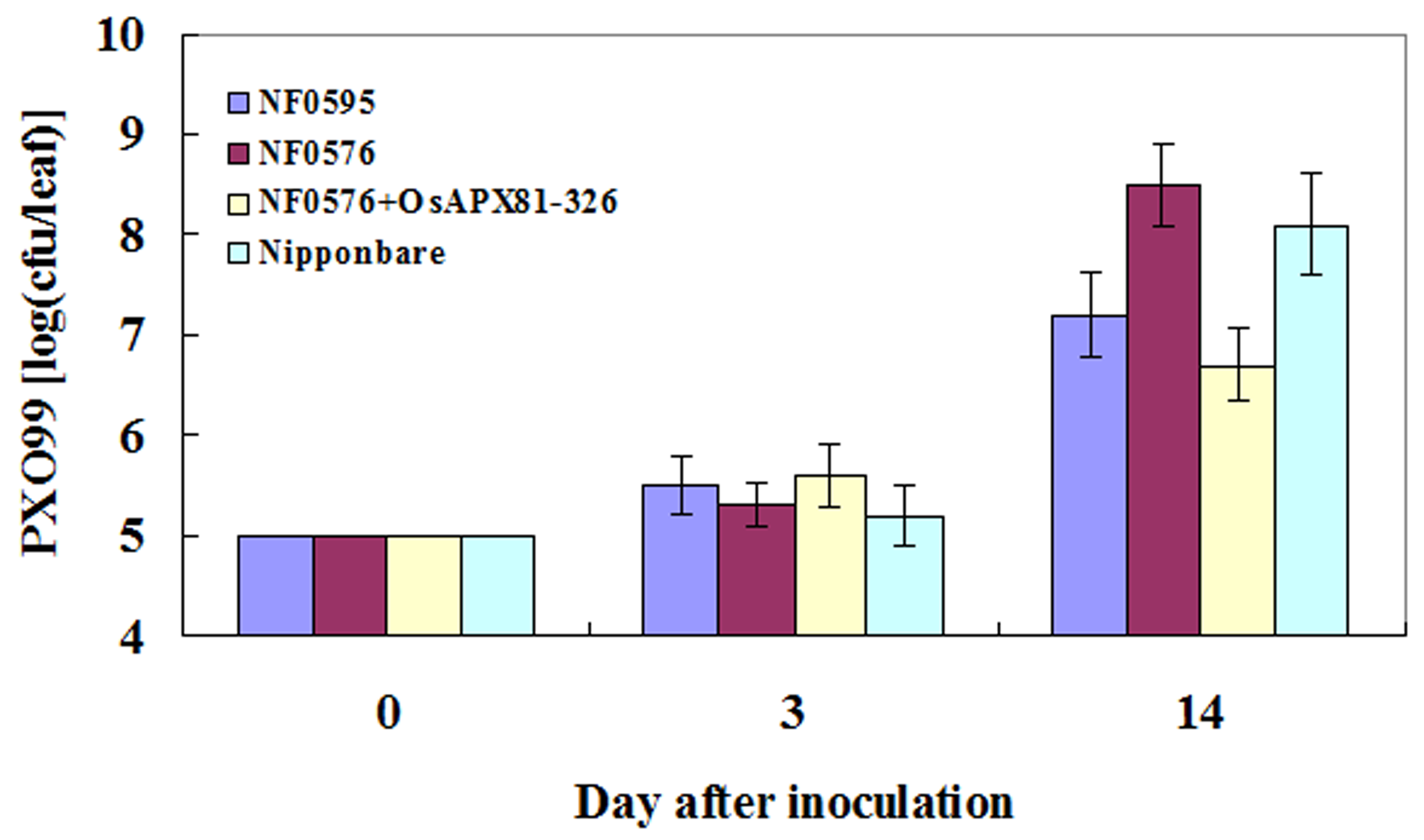


A


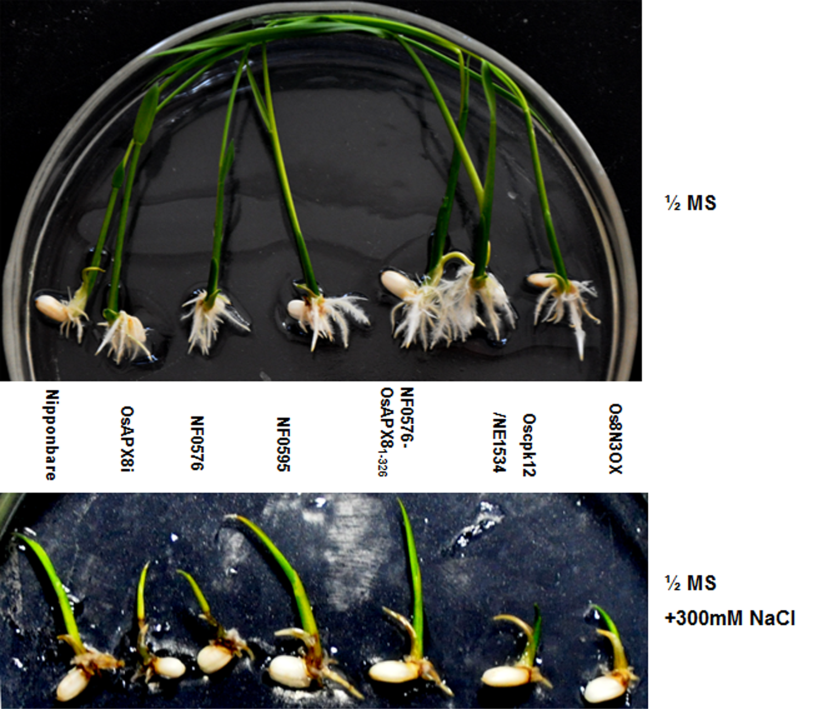


C

Supplementary Figure 7. The functions OsAPX8 in rice tolerance to salt and bacterial blight can be suppressed by Os8N3/Xa13 through the binding of Os8N3/Xa13 with the transmembrane domain of OsAPX8. (a) TOS17 mutants of OsAPX8 showed that its peroxidase domain is important for rice tolerance to PXO99. Photographs were taken 14 days after inoculation with PXO99. Below is the expression of the peroxidase and transmembrane domains of OsAPX8 and Os8N3/Xa13 in these plants when challenged with PXO99 3 days after inoculation. (b) Growth of PXO99 in TOS17 insertion mutants of OsAPX8 and the control plant, Nipponbare. Each point represents three independent replications, and standard deviations are indicated. (c) The growth of OsAPX8 TOS17 mutants under salt stress showed that the peroxidase domain of OsAPX8 is important for it tolerance to salt, while its function can be suppressed by Os8N3/Xa13 through the binding of its transmembrane domain with Os8N3/Xa13. Photographs were taken 10 days after treatment with 300 mM NaCl.


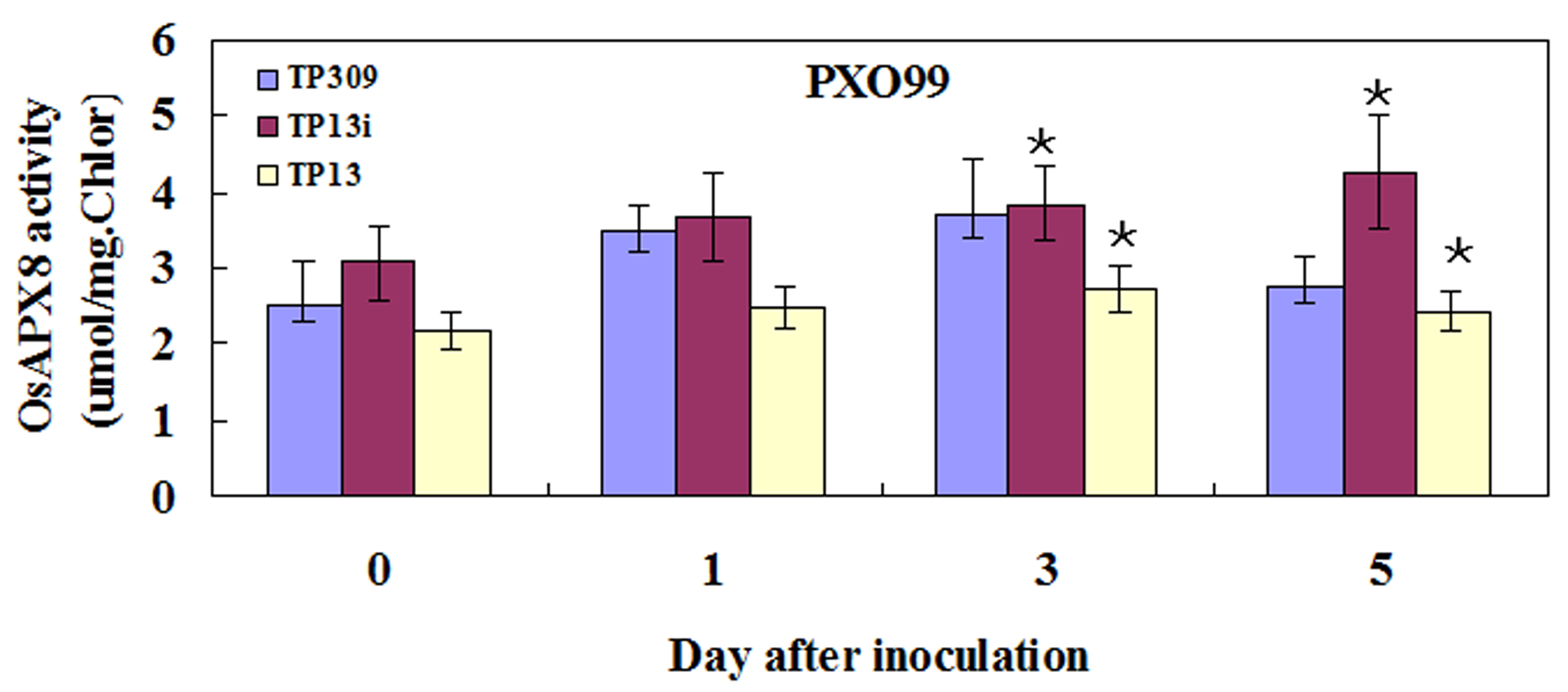


A


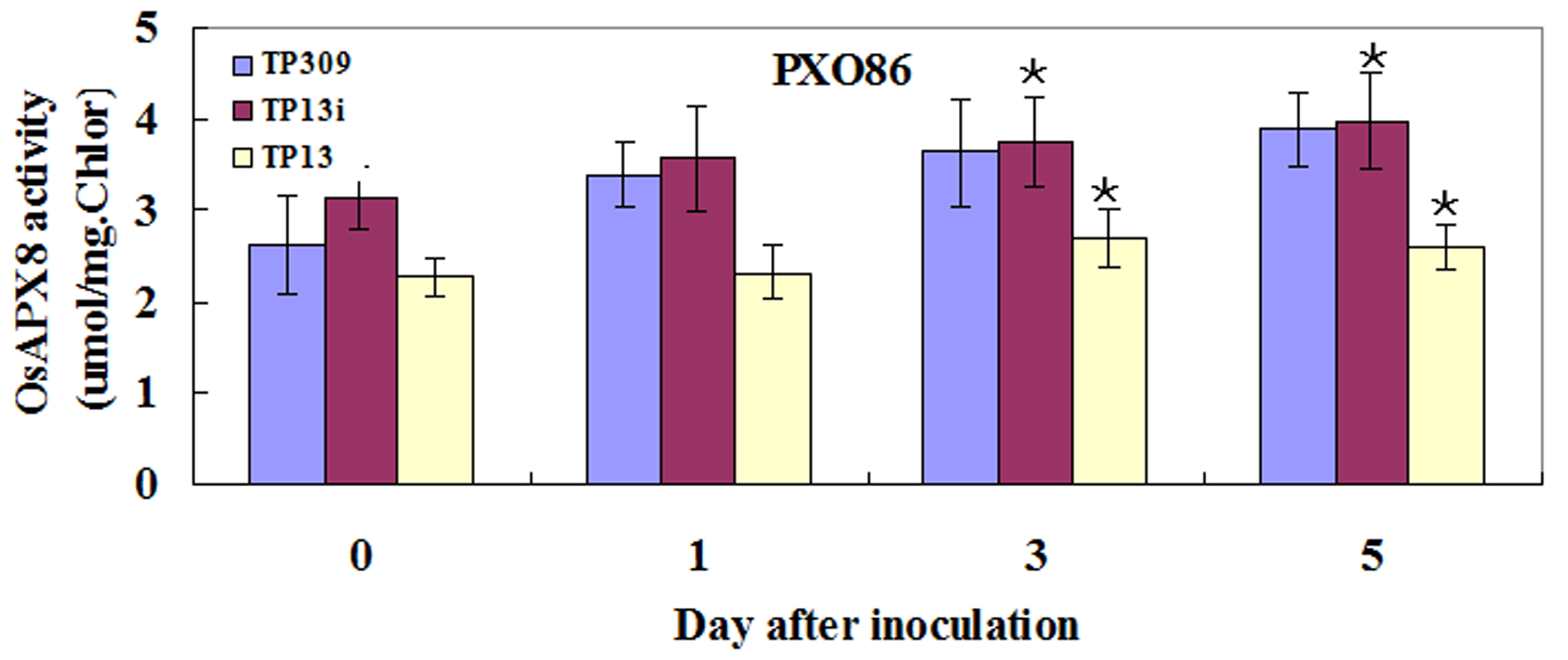


B

Supplementary Figure 8. OsAPX8 activity is negatively correlated with Os8N3/Xa13. (a) OsAPX8 activity increased in TP13i plants when challenged with PXO99, unlike that of TP309, which increased in the early stage and then decreased at 5 DAI. In TP13 plants, this activity was suppressed. (b) OsAPX8 activity increased in TP13i and TP309 plants when challenged with PXO86. Similarly, this activity was suppressed in TP13. Bars are mean values ±SE of three independent experiments.

Supplementary Table 1 Germination rate of transgenic rice seeds under salt stress (%) ^a^


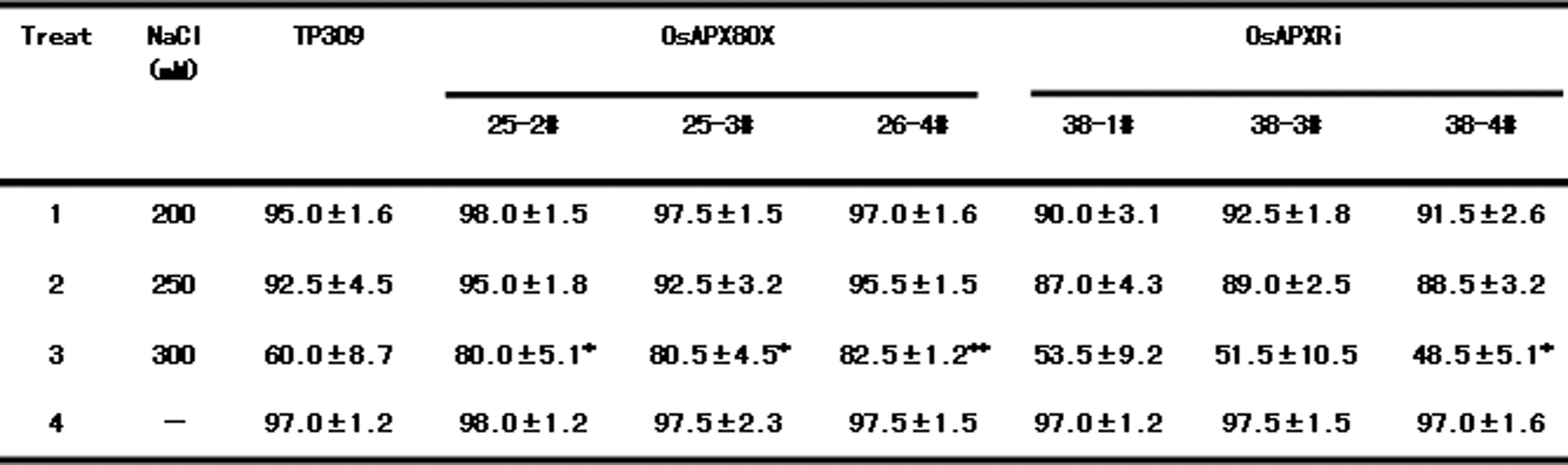


^a^Means ± SD (standard deviation);* and ** significance at the 5% and 1% levels according to Student’s t test.

Supplementary Table 2 Primers for quantitative and semi-quantitative reverse transcriptase PCR.

| Gene | Forward primer(5’→3’) | Reverse primer(5’→3’) | Size(bp) |
| --- | --- | --- | --- |
| OsAPX1 | CTCGAGCTACAAGGAGGCCCACC | CTCAGCCGCATTTCATACCAACAC | 185 |
| OsAPX2 | CTCGAGCCAAGTGACAAAGCCCTC | CTCGAGAAGGCGCAAAATACAAATC | 198 |
| OsAPX3 | GAGCCCAGCTCAGATAGCTTCTTG | GAGTGACAAGGCATTGTTGGAAG | 228 |
| OsAPX4 | GTACCTCGAGCAGCTGCAGCAACAG | GTACCTCGAGCAGCTGCAGCAACAG | 180 |
| OsAPX5 | GAGAGGGCAATCTTGGACATCTG | GGTACCTCGAGGATCAAACTTTG | 215 |
| OsAPX6 | CTCGAGAGGGCAATCTTGGACATC | CCTCGAGGATCAAACTTTGCTCC | 227 |
| OsAPX7 | CTAAACTGAGCAATCTGGGTGC | CGAGGACTCGTGGTCAGGAAAAGC | 193 |
| OsAPX8 | CGAGGCTGCGAAATACTCCTACG | CGAGAGGAGGTCATCAGACCATCG | 197 |
| Actin | ATGCTTCTAATTCTTCGGACC | CCAAAATCACAAGTGAGAACC | 197 |
| LOC_Os02g 02400.1 | CTTCGGCGTCAACACCTACACC | CCTACCTGGACGAACAGCTTCC | 210 |
| LOC_Os03g 03910.1 | TGGTGATTGCCAAGGAGAAC | CCAGTTTCTGACCCAGAGACC | 189 |
| LOC_Os06g 51150.1 | ACAACCACCACGATGGCTCC | CCTGGCAGGGTCAAATGACC | 206 |
| LOC_Os01g 16152.1 | GATGGGCAGAAAAGCTACAGG | GAAACCTTGAAGTCAGAAGG | 202 |
| LOC_Os01g 48420.1 | AGCATGTGAAATTCCTTGCC | TTGAGGATCTCCTCAGCACC | 193 |
| [LOC_Os02g 09940.1](http://rice.plantbiology.msu.edu/cgi-bin/ORF_infopage.cgi?orf=LOC_Os02g09940.1) | GTGCTCCTCCTCTCCGACGG | GGCGCTGCTGGTGGTGAAGG | 180 |
| LOC_Os02g 33450.1 | GGTGTCTTGATCCCTGACCAG | GGTCAGGCTTCATCGACTTG | 217 |
| LOC_Os04g 33970.1 | CAGCATTCTACCATTAACAACC | CAAAGTACTCCTTGCTGTCC | 172 |
| LOC_Os06g 09610.1 | GAGTTCAAGAAGAAGTACAAG | GGCTCTGGAGGATCTTGAGG | 215 |
| LOC_Os06g 42000.1 | ACGCGACGCTCTCCTACTTC | AACGCGTCGTTGACGGAGAC | 217 |
| LOC_Os07g 15670.1 | CTTTTCGAGCCGTCTGCACGC | CCGACACTGGGTTTTGGCAC | 199 |
| LOC_Os11g 18170.1 | ATTGAGCAAACAGTTTGTACC | AACAACCTTGCCATCTTTACC | 183 |
| LOC_Os06g 08670.1 | GCTTGTACAAGGTTTAAAGCTG | GAATGGTGAAGTTGTAGGTGGG | 201 |
| LOC_Os02g 44500.1 | TAATGGTGACAACACTGCACCC | CAGCTTCTTGATGTCCTTCTCC | 178 |
| LOC_Os04g 46960.1 | TAGCATCAAGTGGAACTTCTCC | CCAAGCAGCTTCTTGATATCC | 204 |
| LOC_Os03g 24380.1 | TCAGAAGGTGCGTGTAAATGG | GTCTTTCTCAAAAGACAAGGG | 181 |
| [LOC_Os03g 11960](http://rice.plantbiology.msu.edu/cgi-bin/ORF_infopage.cgi?orf=LOC_Os03g11960).1 | ACCTGGGAAACATAGTAGCC | AGTCCAATGATACCGCATCC | 205 |
| [LOC_Os03g 22810](http://rice.plantbiology.msu.edu/cgi-bin/ORF_infopage.cgi?orf=LOC_Os03g22810).1 | TGGAGCACCAGAAGATGAGAC | CGTTTCCGGTGGTCTTGCTC | 206 |
| [LOC_Os04g 48410](http://rice.plantbiology.msu.edu/cgi-bin/ORF_infopage.cgi?orf=LOC_Os04g48410).1 | ACTAGAAGCTGGAGAGAAGG | AATGGTGACACCATCACAGG | 202 |
| [LOC_Os05g 25850](http://rice.plantbiology.msu.edu/cgi-bin/ORF_infopage.cgi?orf=LOC_Os05g25850).1 | GCTGCTTTACAAGGATCTGG | ATGTTGCTCAGGTAGTCTGG | 197 |
| [LOC_Os06g 02500](http://rice.plantbiology.msu.edu/cgi-bin/ORF_infopage.cgi?orf=LOC_Os06g02500).1 | ACCTGGATTATGAGGATCGG | GAGGCATAGCTCTGATTTGG | 192 |
| [LOC_Os06g 05110](http://rice.plantbiology.msu.edu/cgi-bin/ORF_infopage.cgi?orf=LOC_Os06g05110).1 | ATCAGTCCACTTGCACTTGG | GGATATTTGGTTCACCAAGG | 193 |
| [LOC_Os07g 46990](http://rice.plantbiology.msu.edu/cgi-bin/ORF_infopage.cgi?orf=LOC_Os07g46990).1 | TGTCAATGTCTCTGACAGCC | CCCTGGAGTCCGATGATTCC | 171 |
| [LOC_Os08g 44770](http://rice.plantbiology.msu.edu/cgi-bin/ORF_infopage.cgi?orf=LOC_Os08g44770).1 | CCTGGGAAACATTGTTGCC | AGCATTTCCAGTACTGAGAC | 172 |
| OsHSR203J | CCGACTTCTCGCGGGTGTTCC | CAGCACCTCCACGTCCTTGCC | 419 |
| OsHin1R | CAGTCCACCGAGGCATCCACC | ACAGCAGTAAGTAAGCCGTACC | 530 |

Supplementary Table 3 Subcellular localization prediction of Os8N3 with WoLF PSORT and Chlorop v1.1

1. **Subcellular localization prediction of Os8N3 with WoLF PSORT (**[**http://www.genscript.com/results/143012805716365.html**](http://www.genscript.com/results/143012805716365.html)**)**

**queryProtein WoLFPSORT prediction chlo: 11, mito: 1, extr: 1**

[PSORT features and traditional PSORTII prediction](http://www.genscript.com/results/143012805716365.PSORTverboseOutput.html#queryProtein)

| 14 Nearest Neighbors | | | | |
| --- | --- | --- | --- | --- |
| **id** | **site** | **distance** | **identity** | **comments** |
| MDHM_CHLRE | mito | 395.161 | [14.1711%](http://www.genscript.com/results/143012805716365.alignment1.html#MDHM_CHLRE) | [[Uniprot]](http://www.uniprot.org/entry/Q42686) SWISS-PROT45:Mitochondrial matrix. |
| GGPP_CAPAN | chlo | 440.144 | [13.5135%](http://www.genscript.com/results/143012805716365.alignment1.html#GGPP_CAPAN) | [[Uniprot]](http://www.uniprot.org/entry/P80042) SWISS-PROT45:Chloroplast. |
| PSY_ARATH | chlo | 446.905 | [11.8483%](http://www.genscript.com/results/143012805716365.alignment1.html#PSY_ARATH) | [[Uniprot]](http://www.uniprot.org/entry/P37271) SWISS-PROT45:Chloroplast. |
| PGKH_TOBAC | chlo | 450.723 | [18.0873%](http://www.genscript.com/results/143012805716365.alignment1.html#PGKH_TOBAC) | [[Uniprot]](http://www.uniprot.org/entry/Q42961) SWISS-PROT45:Chloroplast. |
| PGKH_ARATH | chlo | 456.715 | [19.4561%](http://www.genscript.com/results/143012805716365.alignment1.html#PGKH_ARATH) | [[Uniprot]](http://www.uniprot.org/entry/P50318) SWISS-PROT45:Chloroplast. |
| IF2C_ARATH | chlo | 465.429 | [9.25197%](http://www.genscript.com/results/143012805716365.alignment1.html#IF2C_ARATH) | [[Uniprot]](http://www.uniprot.org/entry/Q9SHI1) SWISS-PROT45:Chloroplast. |
| CHLI_TOBAC | chlo | 471.121 | [15.7277%](http://www.genscript.com/results/143012805716365.alignment1.html#CHLI_TOBAC) | [[Uniprot]](http://www.uniprot.org/entry/O22436) SWISS-PROT45:Chloroplast stroma. |
| E13H_TOBAC | extr | 473.4 | [13.2743%](http://www.genscript.com/results/143012805716365.alignment1.html#E13H_TOBAC) | [[Uniprot]](http://www.uniprot.org/entry/P36401) SWISS-PROT45:Extracellular. |
| HMA3_HORVU | chlo | 473.887 | [13.4579%](http://www.genscript.com/results/143012805716365.alignment1.html#HMA3_HORVU) | [[Uniprot]](http://www.uniprot.org/entry/O65796) SWISS-PROT45:Chloroplast. |
| HMA1_HORVU | chlo | 481.204 | [14.0417%](http://www.genscript.com/results/143012805716365.alignment1.html#HMA1_HORVU) | [[Uniprot]](http://www.uniprot.org/entry/Q42843) SWISS-PROT45:Chloroplast. |
| PGKH_WHEAT | chlo | 482.482 | [15.8333%](http://www.genscript.com/results/143012805716365.alignment1.html#PGKH_WHEAT) | [[Uniprot]](http://www.uniprot.org/entry/P12782) SWISS-PROT45:Chloroplast. |
| PORA_HORVU | chlo | 509.04 | [15.7216%](http://www.genscript.com/results/143012805716365.alignment1.html#PORA_HORVU) | [[Uniprot]](http://www.uniprot.org/entry/P13653) SWISS-PROT45:Chloroplast. |
| At2g45300.1 | chlo | 509.245 | [15.9615%](http://www.genscript.com/results/143012805716365.alignment1.html#At2g45300.1) | [[Arath]](http://arabidopsis.org/servlets/mapper?value=At2g45300.1&action=search) |
| At3g55120.1 | E.R._vacu | 509.776 | [13.355%](http://www.genscript.com/results/143012805716365.alignment1.html#At3g55120.1) | [[Arath]](http://arabidopsis.org/servlets/mapper?value=At3g55120.1&action=search) |

| Normalized Feature Values | | | | | | | | | | | | | | | | | | | | | | | | | | |
| --- | --- | --- | --- | --- | --- | --- | --- | --- | --- | --- | --- | --- | --- | --- | --- | --- | --- | --- | --- | --- | --- | --- | --- | --- | --- | --- |
| **id** | **site** | **iPSORT** | | [**PSORT Features**](http://www.genscript.com/WoLFPSORTdoc/psortFeatureDescriptions.html) | | | | | | | | | | | | | | **Amino Acid Content** | | | | | | | | **Misc.** |
|  |  | [**MxHy1_30**](http://www.genscript.com/WoLFPSORTdoc/maxHydropathy0_29_12Description.html) | [**Mx-1_20**](http://www.genscript.com/WoLFPSORTdoc/maxNegativeCharge0_19_12Description.html) | [**dna**](http://www.genscript.com/WoLFPSORTdoc/dnaDescription.html) | [**erl**](http://www.genscript.com/WoLFPSORTdoc/erlDescription.html) | [**m1b**](http://www.genscript.com/WoLFPSORTdoc/m1bDescription.html) | [**m3a**](http://www.genscript.com/WoLFPSORTdoc/m3aDescription.html) | [**mNt**](http://www.genscript.com/WoLFPSORTdoc/mNtDescription.html) | [**mip**](http://www.genscript.com/WoLFPSORTdoc/mipDescription.html) | [**mit**](http://www.genscript.com/WoLFPSORTdoc/mitDescription.html) | [**nuc**](http://www.genscript.com/WoLFPSORTdoc/nucDescription.html) | [**pox**](http://www.genscript.com/WoLFPSORTdoc/poxDescription.html) | [**psg**](http://www.genscript.com/WoLFPSORTdoc/psgDescription.html) | [**rib**](http://www.genscript.com/WoLFPSORTdoc/ribDescription.html) | [**rnp**](http://www.genscript.com/WoLFPSORTdoc/rnpDescription.html) | [**tms**](http://www.genscript.com/WoLFPSORTdoc/tmsDescription.html) | [**yqr**](http://www.genscript.com/WoLFPSORTdoc/yqrDescription.html) | [**A**](http://www.genscript.com/WoLFPSORTdoc/AcontDescription.html) | [**C**](http://www.genscript.com/WoLFPSORTdoc/CcontDescription.html) | [**Q**](http://www.genscript.com/WoLFPSORTdoc/QcontDescription.html) | [**H**](http://www.genscript.com/WoLFPSORTdoc/HcontDescription.html) | [**I**](http://www.genscript.com/WoLFPSORTdoc/IcontDescription.html) | [**L**](http://www.genscript.com/WoLFPSORTdoc/LcontDescription.html) | [**S**](http://www.genscript.com/WoLFPSORTdoc/ScontDescription.html) | [**V**](http://www.genscript.com/WoLFPSORTdoc/VcontDescription.html) | [**length**](http://www.genscript.com/WoLFPSORTdoc/lengthDescription.html) |
| queryProtein | chlo? | 92 | 20 | 47 | 50 | 48 | 47 | 49 | 156 | 49 | 83 | 49 | 92 | 49 | 50 | 95 | 44 | 95 | 63 | 16 | 16 | 55 | 90 | 43 | 99 | 43 |
| MDHM_CHLRE | mito | 92 | 20 | 47 | 50 | 48 | 47 | 49 | 96 | 57 | 30 | 49 | 100 | 49 | 50 | 84 | 94 | 96 | 63 | 44 | 20 | 22 | 51 | 53 | 97 | 54 |
| GGPP_CAPAN | chlo | 76 | 20 | 47 | 50 | 48 | 47 | 49 | 84 | 70 | 79 | 49 | 84 | 49 | 50 | 39 | 44 | 89 | 72 | 28 | 64 | 65 | 88 | 14 | 66 | 54 |
| PSY_ARATH | chlo | 88 | 20 | 47 | 50 | 48 | 47 | 49 | 71 | 53 | 88 | 49 | 85 | 49 | 50 | 39 | 44 | 69 | 56 | 41 | 3 | 19 | 89 | 73 | 70 | 64 |
| PGKH_TOBAC | chlo | 51 | 20 | 47 | 50 | 48 | 47 | 49 | 98 | 82 | 76 | 49 | 93 | 49 | 50 | 39 | 44 | 91 | 17 | 3 | 36 | 43 | 92 | 69 | 89 | 72 |
| PGKH_ARATH | chlo | 86 | 45 | 47 | 50 | 48 | 47 | 49 | 98 | 86 | 76 | 49 | 87 | 49 | 50 | 39 | 44 | 85 | 12 | 3 | 36 | 62 | 84 | 64 | 96 | 71 |
| IF2C_ARATH | chlo | 89 | 20 | 47 | 50 | 48 | 47 | 49 | 85 | 47 | 83 | 49 | 79 | 49 | 50 | 39 | 44 | 79 | 24 | 37 | 16 | 50 | 22 | 68 | 95 | 96 |
| CHLI_TOBAC | chlo | 83 | 20 | 47 | 50 | 48 | 47 | 49 | 99 | 85 | 71 | 49 | 78 | 49 | 50 | 39 | 44 | 55 | 43 | 48 | 34 | 75 | 85 | 53 | 84 | 64 |
| E13H_TOBAC | extr | 96 | 20 | 47 | 50 | 48 | 47 | 49 | 92 | 52 | 30 | 49 | 95 | 49 | 50 | 84 | 94 | 60 | 16 | 62 | 15 | 71 | 78 | 52 | 55 | 48 |
| HMA3_HORVU | chlo | 85 | 20 | 47 | 50 | 48 | 47 | 49 | 82 | 77 | 65 | 49 | 93 | 49 | 50 | 39 | 44 | 90 | 71 | 39 | 52 | 40 | 52 | 58 | 86 | 77 |
| HMA1_HORVU | chlo | 79 | 71 | 47 | 50 | 48 | 47 | 49 | 78 | 65 | 65 | 49 | 85 | 49 | 50 | 39 | 44 | 92 | 66 | 24 | 53 | 50 | 65 | 54 | 83 | 77 |
| PGKH_WHEAT | chlo | 75 | 71 | 47 | 50 | 48 | 47 | 49 | 97 | 70 | 30 | 49 | 84 | 49 | 50 | 39 | 44 | 97 | 17 | 5 | 19 | 34 | 93 | 46 | 86 | 71 |
| PORA_HORVU | chlo | 79 | 71 | 47 | 50 | 48 | 47 | 49 | 98 | 47 | 71 | 49 | 89 | 49 | 50 | 39 | 44 | 91 | 35 | 32 | 60 | 3 | 74 | 86 | 68 | 59 |
| At2g45300.1 | chlo | 33 | 45 | 47 | 50 | 48 | 47 | 49 | 96 | 89 | 86 | 49 | 98 | 49 | 50 | 39 | 44 | 56 | 72 | 20 | 16 | 52 | 71 | 75 | 83 | 76 |
| At3g55120.1 | E.R._vacu | 67 | 45 | 47 | 50 | 48 | 47 | 49 | 83 | 57 | 30 | 49 | 91 | 49 | 50 | 39 | 44 | 33 | 46 | 18 | 13 | 58 | 58 | 86 | 88 | 31 |

**B.** Chloroplast transit peptides prediction of Os8N3 with Chlorop v1.1 (http://www.cbs.dtu.dk/services/ChloroP/)

chlorop v1.1 prediction

**--------------------------------------------------------------------------------------------**

**Os8N3 Length Score cTP CS- cTP-**

**score length**

**--------------------------------------------------------------------------------------------**

**Sequence 307 0.539 YES 1.756 14**

**--------------------------------------------------------------------------------------------**
